# Multiple roles of the transcription factor AtMYBR1/AtMYB44 in ABA signaling, stress responses, and leaf senescence

**DOI:** 10.1186/1471-2229-13-192

**Published:** 2013-11-28

**Authors:** Masrur R Jaradat, J Allan Feurtado, Daiqing Huang, Yongquan Lu, Adrian J Cutler

**Affiliations:** 1 Plant Biotechnology Institute, National Research Council of Canada, 110 Gymnasium Place, Saskatoon S7N 0W9, Canada; 2 Zhejiang Agriculture and Forestry University, Hangzhou 311300, China

**Keywords:** ABA, Drought stress, Transcription factor, PYL8, Senescence

## Abstract

**Background:**

The transcription factor At*MYBR1* (*MYB44*) is a member of the MYB family of transcription factors and is expressed throughout the plant life cycle and especially in senescing and wounded leaves. It has previously been shown to be involved in responses to abiotic stress and is regulated by phosphorylation.

**Results:**

When *MYBR1* was over-expressed under the control of the constitutive 35S promoter in *Arabidopsis thaliana* (Ox*MYBR1*), leaf senescence was delayed. In contrast loss-of-function *mybr1* plants showed more rapid chlorophyll loss and senescence. The *MYBR1* promoter strongly drove *β-GLUCURONIDASE* reporter gene expression in tissues immediately after wounding and many wounding/pathogenesis genes were downregulated in Ox*MYBR1*. Ox*MYBR1* plants were more susceptible to injury under water stress than wildtype, which was correlated with suppression of many ABA inducible stress genes in Ox*MYBR1*. Conversely, *mybr1* plants were more tolerant of water stress and exhibited reduced rates of water loss from leaves. MYBR1 physically interacted with ABA receptor PYR1-LIKE8 (PYL8) suggesting a direct involvement of MYBR1 in early ABA signaling. *MYBR1* appears to exhibit partially redundant functions with At*MYBR2* (*MYB77*) and double *mybr1* X *mybr2* mutants exhibited stronger senescence and stress related phenotypes than single *mybr1* and *mybr2* mutants.

**Conclusions:**

*MYBR1* is a negative regulator of ABA, stress, wounding responses and blocks senescence. It appears to have a homeostatic function to maintain growth processes in the event of physical damage or stress.

## Background

In order to acclimate and protect themselves, plants translate environmental challenges such as drought, waterlogging, extreme temperatures, soil salinity, wounding, and pathogen attack into internal signals through hormones, second messengers, and transcription factors (TFs). The phytohormone abscisic acid (ABA) regulates abiotic stress responses and other vital processes in plant growth and development, especially during seed maturation reviewed in [1]. Mutant plants with altered ABA biosynthesis, perception or response have been crucial in deciphering the various components involved in ABA responses.

Recently, a family of 14 novel START domain proteins, named as PYR/PYL/RCARs (PYRABACTIN RESISTANCE/PYR1 LIKE/REGULATORY COMPONENT OF ABA RECEPTOR) has been identified as intracellular ABA receptors that interact with and inhibit several protein phosphatase 2Cs (PP2Cs) including ABA INSENSITIVE1 and 2 (ABI1, ABI2), HOMOLOGY TO ABI1 (HAB1), and PP2CA [2-4]. Such phosphatases are negative regulators of ABA signaling. A recent model for ABA signaling, based on several independent crystallographic studies for example [5], proposes that in the presence of ABA, receptors of the PYR/PYL/RCAR family bind to PP2Cs which in turn release inhibition on a subfamily of SNF1-RELATED PROTEIN KINASE2 (SnRK2) kinases. These kinases then phosphorylate and subsequently activate transcription factors including ABA RESPONSIVE ELEMENTS-BINDING FACTOR (ABF)/ ABA RESPONSIVE ELEMENTS-BINDING PROTEIN (AREB)-type bZIP TFs. ABFs in turn bind to ABA-responsive promoter elements (ABRE) to activate ABA-responsive gene expression.

The molecular basis of adaptive responses to abiotic stresses such as low temperature, desiccation and salinity, has been elucidated by identifying genes such as *RESPONSIVE TO DEHYDRATION* (*RD*), *KYKNA-INDUSOITU* (*KIN*; Finnish for cold-induced), *RESPONSIVE TO ABA* (*RAB*)*, COLD-REGULATED* (*COR*), *LOW-TEMPERATURE-INDUCED* (*LTI*), and *DROUGHT-INDUCED* (*DI*). Manipulation of many of these genes resulted in plants with improved tolerance to drought, salt, cold and freezing reviewed in [6]. Molecular and genetic studies suggest that ABA-dependent and –independent pathways operate in abiotic stresses and ABA-dependent pathways are predominant in drought stress responses [7,8].

Environmental factors such as drought, extreme temperature and pathogen infection as well as endogenous factors including age affect the onset and progression of leaf senescence reviewed in [9]. Unlike abscission and dehiscence, leaf senescence is a specialized form of programmed cell death, which is a genetically regulated process of slow cell death of the entire leaf and is preceded by the reallocation of nitrogen, phosphorus, and metals to other parts of the plant. An early manifestation of senescence in leaves is loss of chlorophyll. Leaf mesophyll cells start to senesce first, followed by other cell types, and exhibit an incoherent pattern of localized cell death, which eventually spreads to the whole leaf. Senescence is accelerated by ABA, ethylene, jasmonates (JAs), and salicylic acid (SA), and is delayed by cytokinins (CKs) and auxin reviewed in [9]. However, extensive cross talk among these signaling pathways during senescence complicates understanding of the initiation and progression of senescence. Therefore, key components in senescence signaling remain largely unknown.

Senescence is an important aspect of drought responses. Accelerated leaf senescence followed by leaf abscission is triggered by prolonged stress to reduce water loss, remobilize nutrients to young leaves and to enable survival of the plant [10,11].

The MYB family TFs comprises around 180 genes in *Arabidopsis* and is the largest TF gene family reviewed in [12]. MYB proteins contain a conserved DNA-binding MYB domain of about 52 amino acids, and are classified into three subfamilies based on the presence of one, two or three MYB domains reviewed in [13]. The plant specific and largest MYB family consists of R2R3-type factors which contain two repeats and comprise 125 genes in *Arabidopsis. R2R3-MYB* genes are involved in various plant-specific processes such as regulation of secondary metabolism, modulation of development, determination of cell fate and identity and responses to environmental factors and hormone. The gene further characterized in this paper, At*MYBR1/MYB44* (*R2R3 MYB*) was weakly induced by 24 h treatment with ABA but strongly induced by the hyperactive ABA analog (+)-8′ acetylene ABA (PBI425) [14]. Most ABA-regulated genes are similarly regulated by water stress, however *MYBR1* was selected for further functional characterization because its expression was paradoxically repressed by drought and elevated by re-watering [8] suggesting a novel role in ABA signaling. Jung et al. [15] reported that over-expression of *MYBR1* increased stress tolerance but unexpectedly repressed many known stress-related genes. Subsequent studies have revealed that this gene is regulated by a Mitogen-Activated Protein Kinase (MAPK) cascade. Following stress treatment, MITOGEN-ACTIVATED PROTEIN KINASE (MPK3) is activated and phosphorylates the bZIP TF VirE2-INTERACTING PROTEIN 1 (VIP1), which then rapidly activates the expression of *MYBR1* and other stress genes through promoter binding [16]. Further studies have shown that MYBR1 interacts directly with, and is phosphorylated by, MPK3 at ser145 [17] and possibly ser53 [18] and that the ser145 phosphorylation is required for MYBR1 function [17]. In this study, we functionally characterized the At*MYBR1* TF by studying an *Arabidopsis* T-DNA insertion mutant *mybr1* and overexpression lines of At*MYBR1* (Ox*MYBR1*). We show that *MYBR1* down regulates many ABA responsive genes including those involved in abiotic stresses and negatively regulates drought responses and senescence. Moreover, direct involvement of MYBR1 in early ABA signaling is suggested by our observation that MYBR1 protein interacts with PYL8, an ABA receptor.

## Results

### AtMYBR1 represses genes induced by a hyperactive ABA analog

We showed in a previous study that At*MYBR1* was induced weakly by (+)-ABA and more strongly by 24 h treatment with a hyperactive ABA analog PBI425 ((+)-8′ acetylene ABA) indicating *MYBR1* is likely a component of the ABA signaling pathway [14]. It has been shown previously that PBI425 induces ABA responsive genes almost identically to the natural enantiomer S-(+)-ABA. However, because PBI425 is catabolized much less rapidly than (+)-ABA and accumulates to higher levels in plant tissue [14] it is an effective tool to study weak and transiently expressed ABA-responsive genes such as *ABI1*, *ABI2*, *LTI30, KNAT4* and *MYBR1* itself [8,14,19]. Therefore we used PBI425 to define the role of *MYBR1* in ABA signaling.

In addition to using PBI425 to study the function of At*MYBR1*, we generated transgenic *Arabidopsis* 35S_pro_:*MYBR1* plants (Ox*MYBR1*). After kanamycin selection, three lines with single inserts were selected and their homozygous progeny plants were grown as experimental materials. The level of overexpression of *MYBR1* was 23-fold in gain-of-function Ox*MYBR1* line 42–6, 12-fold in line 31–3 and 11-fold in line 1–7 and was undetectable by qPCR in loss-of-function *mybr1*[14]. To reduce the likelihood of identifying phenotypic artifacts due to mis-expression, the phenotypes of all the overexpression lines were compared for qualitative consistency throughout the experiments.

**Figure 1:**
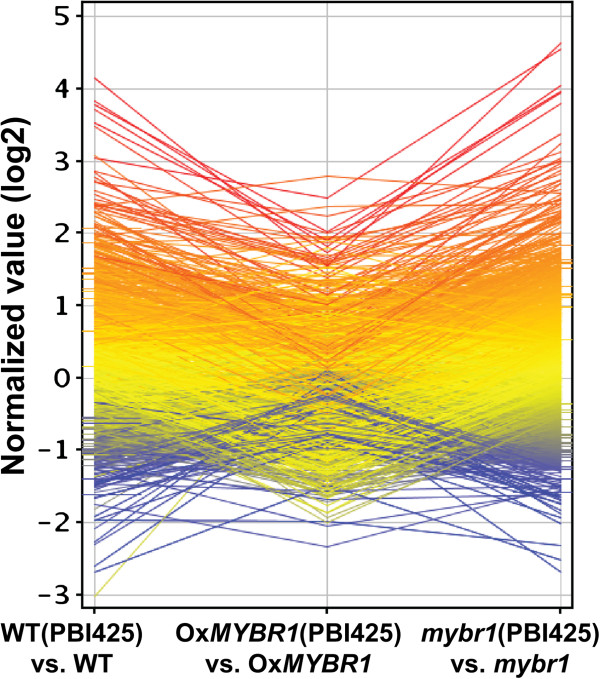
**Gain of At*MYBR1 *function results in suppression of ABA induced changes in gene expression.** The effects of PBI425 on gene expression (obtained from microarray comparisons) are compared in the three genetic backgrounds. Changes induced by PBI425 (both induction and repression) in WT and *mybr1* backgrounds are reduced in the Ox*MYBR1* background.

We compared gene expression in different genotypes using *Arabidopsis* oligoarrays representing a comprehensive set of approximately 26,000 expressed genes. The comparisons were: (i) genotype comparisons of untreated plants: Ox*MYBR1* (42–6) or *mybr1* versus WT, (ii) genotype comparisons after PBI425 treatment: Ox*MYBR1* (42–6) or *mybr1* versus WT, and (iii) effect of PBI425 treatments on each genotype: Ox*MYBR1*, *mybr1* and WT treated with PBI425 versus the same genotype without the treatment. The experimental design is illustrated in Additional file 1: Figure S1 online. Samples were treated with PBI425 for 24 h on the basis that the accumulation and effects of PBI425 on gene expression was maximum at 24 h [8]. The total numbers of differentially expressed genes are listed in Table 1 and the gene lists and data may be found in Additional file 2: Table S1. There were a total of 1507 differentially regulated genes from all comparisons. In the absence of PBI425 treatment, comparisons of Ox*MYBR1* or *mybr1* vs. WT yielded a very small number of differentially regulated genes (Table 1). Treatment with PBI425 greatly increased numbers of differentially expressed genes and revealed differences between genotypes. Analysis of the direct effect of PBI425 on gene expression showed that *MYBR1* represses expression of many genes induced by PBI425 in WT (and *mybr1*) in terms of both total numbers (Table 1) and expression ratios (Figure 1, Additional file 2: Table S1).

**Table 1:** Number of significantly up- and down regulated genes using a threshold change in expression of 1.5 Fold and a P-value cut-off: ≤ 0.05

**Hybridization**	**Up**	**Down**	**Unchanged**
*mybr1*(PBI425) vs. *mybr1*	448	452	607
WT(PBI425) vs. WT	417	438	652
Ox*MYBR1*(PBI425) vs. Ox*MYBR1*	180	246	1081
Ox*MYBR1*(PBI425) vs. WT(PBI425)	88	420	999
Ox*MYBR1* vs. WT	35	86	1386
*mybr1*(PBI425) vs. WT(PBI425)	11	1	1495
*mybr1* vs. WT	0	2	1505

It is noteworthy that there were very few differentially regulated genes from comparisons of *mybr1* versus WT both with (12 genes) and without (2 genes) PBI425 treatment. This suggests the likelihood that *MYBR1* is functionally redundant with at least one other closely related gene. *MYBR2* (*MYB77*) is reported as the closest homolog of *MYBR1* based on sequence similarities in the C-terminal regions of the respective proteins and lack of homology with other MYB-type proteins [20]. *MYBR2* has been reported to be involved in auxin signal transduction. *MYBR2* over-expression results in reduced root and shoot growth, and root phenotypes in loss-function *mybr2* lines varied with application of auxin [21]. However, its role in ABA responses and stress signaling or whether it acts cooperatively with *MYBR1* remains unclear.

To investigate possible redundant functions of *MYBR1* (MYB44) and *MYBR2 (MYB77)*, gene expression analysis was performed using Agilent microarrays containing 44,000 *Arabidopsis thaliana* reporter sequences. Gene expression was compared between pairs of genotypes treated with PBI425 i) *mybr1* versus *mybr2* and ii) *mybr1* versus *mybr1*x*mybr2*. The number of differentially expressed genes was 56 for *mybr1* vs *mybr2* and 411 for *mybr1* vs *mybr1* x *mybr2* (Additional file 2: Table S1). The increase in differentially expressed genes in the double mutant comparison suggests that *MYBR1* and *MYBR2* act in a synergistic manner However, only six out of 56 genes in the first comparison and 45 out of 411 genes in the second comparison were present in the above mentioned list of 1507 genes differentially expressed in all comparisons.

### AtMYBR1 represses many ABA inducible stress genes

**Figure 2:**
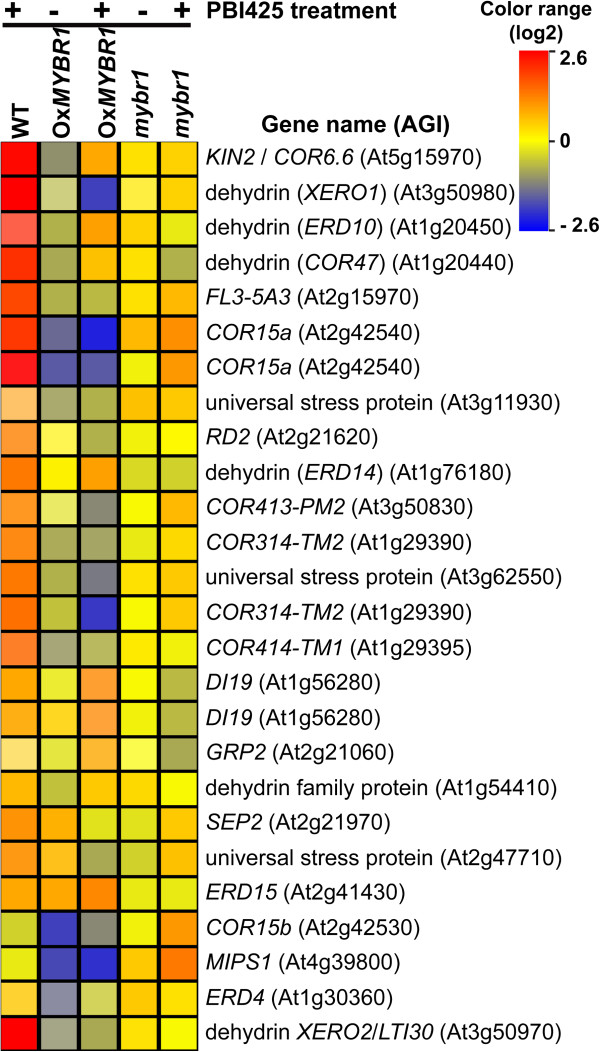
**Expression pattern of 24 stress responsive genes in WT and gain- and loss of At*MYBR1 *function mutant genotypes with and without PBI425.** Comparisons are relative to (i) WT without PBI425 treatment for columns 1, 2 and 4 and (ii) WT with PBI425 treatment for columns 3 and 5 from left to right.

Many stress genes that are highly induced by ABA, are repressed by *MYBR1* (Figure 2). However, since *MYBR1* did not appear to repress all PBI425 induced genes, we examined more closely the gene expression patterns affected by *MYBR1* and PBI425. In this analysis, we added 32 statistically significant genes to the total gene list of Table 1 for detailed analysis and interpretation. These 32 genes were not listed in Table 1 since their fold change was below the 1.5 ratio threshold. However, changes in expression of these genes were either verified by qPCR and direct spot visualization in BASE or were present in our comparative analysis of our microarray data with data published by van der Graaff et al. [22].

Several distinct gene regulation patterns (classes A - H) were identified by comparing PBI425 treatment and *MYBR1* overexpression (Table 2) and the gene lists and expression data may be found in Additional file 3: Table S4. We excluded the comparison of *mybr1* vs. WT from Table 2 because of the small number of differentially expressed genes. Similarly, we also excluded microarray data of *mybr2* from comparisons *mybr1*(PBI425) versus *mybr2*(PBI425) and *mybr1*(PBI425) versus *mybr1*x*mybr2*(PBI425). Many well-characterized abiotic stress responsive genes were grouped in classes A, F and G, of which 278 genes (class A, representing repression of ABA responses by *MYBR1*) were repressed by *MYBR1*, 112 genes (class F, ABA-like activation) were activated by *MYBR1* and 254 genes (class G, representing ABA-specific activation) were unaffected by *MYBR1*. Therefore, *MYBR1* represses a subset of about 43% of ABA-responsive genes. It has been suggested that stress responsive genes are under regulation by both ABA-dependent and ABA-independent pathways For a review see [7]. There are four DEHYDRATION RESPONSIVE ELEMENT BINDING PROTEIN1 *(*DREB1*)/*C-REPEAT BINDING FACTOR (CBF) transcription factor genes that have been suggested to mediate ABA independent cold stress responses. Here, *DREB1A*/*CBF3* was induced by PBI425 and repressed by *MYBR1* suggesting that *DREB1A*/*CBF3* is ABA-dependent.

**Table 2:** **Combined effects of ABA (PBI425) treatment and *MYBR1 *** **overexpression on ABA-related gene expression**

**Class**	**Classification of gene regulation type**	**Net effect measured**					**Differentially expressed genes N**^**o**^	**Examples of pathways (bold) / genes (*italic *)**
		**ABA in WT bk**	**ABA in *mybr1 *** **bk**	**ABA in Ox bk**	***MYBR1 *** **Ox in ABA/WT bk**	***MYBR1 *** **Ox in WT bk**		
**A**	Repression of ABA response	↑	↓	↑ but lowered	↓	↓	**278**	**1**^**o **^**metabolism** (*SUS3*; *BMY7*; *GOLS2*; *SIP2*; *ALDH7B4*; *AOX1A*; *LP1*; *LTP3/4*; *PLDδ*; *POP2*; *LKR*; *CORI3*) **2**^**o **^**metabolism** (*4CL1*; *CCoAOMT1*; *OMT1*; *ELI3-1*(*CAD*); *CPISCA*; *CUT1*; *PAP1*); **ABA** (*NCED4*; *ABF3*; *ABI1*; *HVA22D*; *PP2C*); **ethylene** (*ACO* putative; *MBF1C*; *EBF1*); **jasmonates** (*VSP1*; *VSP2*); **redox** (*CAT2*; *FSD1*); **abiotic stress** (*P5CS1*; *COR15a*/*15b*; *FL3-5A3*; *COR413-PM1*; *USP*s; *RCI2B*; *ERD4*/*10*; *COR47*; *XERO2*; *KIN2*(*COR6.6*); *DREB1A*(*CBF3*); *RD29B*; *RD29A*(*COR78*/*LTI78*); *RCI2A*/*2B*; *DI21*; *RD2/22*; *RAB18*); *GPX6*; *BGAL6*; **senescence** (*SAG12*/*13*/*21*/*29*/*102; ERD1*; *APG8a*/*8e*); **cell wall** (*XTH7*; *AGP12*; *EXLA1*); *GSTZ1*; *GSTU7*/*16*; *CYP89A5*/*A6*; *ACP5*; *VIF1*; TFs (*DREB1A; STO*; *STH3*/*LZF1*; *HAP5C*; *HB1*/*7*/*12*; *AGL20*(*SOC1*); *MYB73*; *GT2*; *NFYC3*); H*AB1*/*2*; *LEA14*; *ACD1*; *RD26*; *COR413-PM2*; *COR414-TM1*; *COR314-TM2*; **signaling** (*ROP2*; *GDI1*);
**B**	ABA-like repression	↓	↓	↓	↓	↓	**386**	**Photosynthesis** (**PSI**; **PSII**; *RBCS-1A*/*1B*); *GAPA-2*; **cell wall** (*CSLD5*; *FLA8*; *AGP11*/*13*/*16/20*/*21*; *EXPA1*/*8*/*10*/*15*; *XTH6*); **lipid** (*ACP1*/*3*/*4*; *NMT3*; *CLS*; *SLD1*); *NIA2*; **auxin** (*ILL2*; responsive genes); *DWF1*; 40S and 60S ribosomal protein genes; **biotic stress** (*DAD1*/*2*; *PDF1.1*/*1.2*/*1.2b*/*1.2c*/*1.3*/*2.2*/*2.3*; *TIR*); **senescence** (*SEN1*); TFs (*RAP2.4*; *COL5*/*15*; *GATA5*; *KNAT6*; *WUS*; *BET9*); *SNG1*; **cytokinin** (*ARR4*); **signaling** (*CAM3*; *CDPK6*; *CPK7*; *MKK5*; *GRF6*);
**C**	MYBR1 independent ABA repression	↓	↓	↓	**—**	**—**	**399**	**Photosynthesis** (**PSII**; *RBCS-3B*); **cell wall** (*CSLC4*; *FLA9*; *EXPA3*/*5*/*6*/*11; PME1/3*); **lipid** (*FAD3*/*5*/*7*; *NMT2*; *ATS1*); **auxin** (*PIN4*; *IAA4*; responsive genes); **cytokinin** (*ARR5*/*7*; *IPT2*); **GA** (*GASA4*); 30S, 40S, 50S and 60S ribosomal protein genes; **abiotic/biotic stress** (*ERD3*; *PR5*); **tetrapyrrole biosynthesis**; TFs (*BEE2*; *PRE1*; *HB5*); *TCH3*; *ERD6*; *TIP2*
**D**	ABA independent repression	**—**	**—**	**—**	↓	↓	**171**	**myo-Inositol** (*MIPS1*); **cell wall** (*CESA1*; *AGP1*/*4*/*7*/*15*/*17*; *FLA1*/*2*; *PRP1*; *EXPA7*; *EXPB3*; *XTH9*); **lipid** (*FAD6*; *CER10*); **auxin** (*NIT1*; *ILL1*; *SHY2* (*IAA3*); *ARF8*); **jasmonates** (*LOX2*); **abiotic stress** (*ERD4*); TFs (*NGA1*; *TRY*; *MYBL2*); **protein degradation** (*SCPL2*; *PREP1*; *MMZ1*(*UEV1A*); *UBC1*; *RHA2B*; *PAB2*; *PAC1*; *PAA1*); **signaling** (*GLR3.3*; *RAN3*; *ROP4*; *GRF12*); **transport** (*PIP3B*; *PIP2A*; *AAP2*; *CAX1*; *TGD1*; *PATL1*); **PSII** (*PSBO-2*; *LHCB2.2*/*4.2*);
**E**	Constitutive activation of ABA-repressed responses	↓	↓	↓	↑	↑	**20**	30S and 50S ribosomal protein genes; **ethylene** (*EIL1*; *EFE*/*ACO*); *UBQ1*; *CAM7*;
**F**	ABA-like activation	↑	↑	↑	↑	↑	**112**	*FAD2*; *SEX1*; *HMG1*; *NIT2*; *γ-VPE*; **senescence** (*SEN2* (*CAT3*)); **abiotic/biotic stress** (*ERD14*/*15*; *HSP15.7-CI*; *ERDJ2A*; wound-responsive gene; *PCC1*); **signaling** (*RD20*; *TCH2*; *CAM1*; *RAB2*; *ELF4*); *PP2A-4*;
**G**	MYBR1 independent ABA activation	↑	↑	↑	**—**	**—**	**254**	**myo-Inositol** (*MIOX1*); **major carbohydrate metabolism** (*APL3*; *SUS1*; *RCP1*); **fermentation** (*ADH*; *ALDH2B4*); **cell wall** (*FLA11*; *MERI5B*; *XTR3*; *EXT3*); **lipid** (*DGK1*; *LTP2*; *ACX1*; *MFP2*; *CUT1*; *CER1*); **abiotic stress** (*SEP2*; *KIN1*; *J8*/*20*; *ACD32.1*; *DI19*; *AOC1*; *RD22*/*26*; *ERD7*); **ABA** (*ABA1*; *CYP707A2*); **ethylene** (*ERF4*); TFs (*HAT2*; *HAP3*; *GBF3*; *KNAT4*); **protein degradation** (*RD21A*; *SCPL11*; *CLPX/C*; *L1D*; *UBQ10*; *UBC28*/*30*/*32*); **signaling** (*RAB7B*; *RAN-1*; *MKK9*; *RAFL32*; *PAT1*); *DRM1*; *β-VPE*; **transport** (*AHA3*; *TMT2*; *SUC2*; *AAP1*; *NTP3*; *KUP11*; *GCN5*; *AATP1*);
**H**	ABA independent activation	**—**	**—**	**—**	↑	↑	**27**	*SON1*; *SFP1*; *TGG1*; *TGG2*; *WAK2*; *β-amylase*; *CSLG3*; *ACD6*; *CAM6*;

**Bk**: background; ***mybr1***: loss-of-function of *MYBR1*; **Ox**: overexpressed; **Gene regulation symbols**-- ↑: upregulation; ↓: downregulation; **—**: either ‘not differentially expressed’ or ‘undetectable’.

Many senescence associated genes including *SAG12/13/21/29/102*, *EARLY RESPONSIVE TO DEHYDRATION1 (ERD1)* and *APG8a* are also grouped in class A (downregulated by *MYBR1* but upregulated by PBI425). There were a total of 498 genes that were either upregulated (class F) or down regulated (class B) by *MYBR1* in the same direction as ABA (PBI425). Among the downregulated genes were many associated with photosynthesis and biotic stresses. A total of 198 genes (classes D and H) were regulated by *MYBR1*, but unaffected by PBI425. Among these, several genes involved in jasmonate and auxin action were repressed.

Promoters of many genes associated with drought, cold stress and salinity contain ABA-responsive element (ABRE), ABRE binding factor (ABF) and dehydration responsive element (DRE) [23]. A search for statistically over-represented *cis*-acting motifs present in the promoters of classified genes sets in Table 2 was carried out against AGRIS and PLACE databases using the analysis tools in Athena [24] and the results are summarized in Additional file 1: Table S2. These promoter motifs mainly consisted of four groups. The majority of motifs are associated with ABA. Other motifs are related to stress, light regulation, gibberellins (GA) and circadian clock, suggesting cross talk between these pathways as was also observed previously [14]. There was a general similarity in the presence of ABA and stress motifs in genes that were regulated specifically by ABA and those that were regulated in the opposite direction by *MYBR1*. No significant enrichment was detected in genes that were regulated specifically by *MYBR1*.

### AtMYBR1 reduces drought tolerance

**Figure 3:**
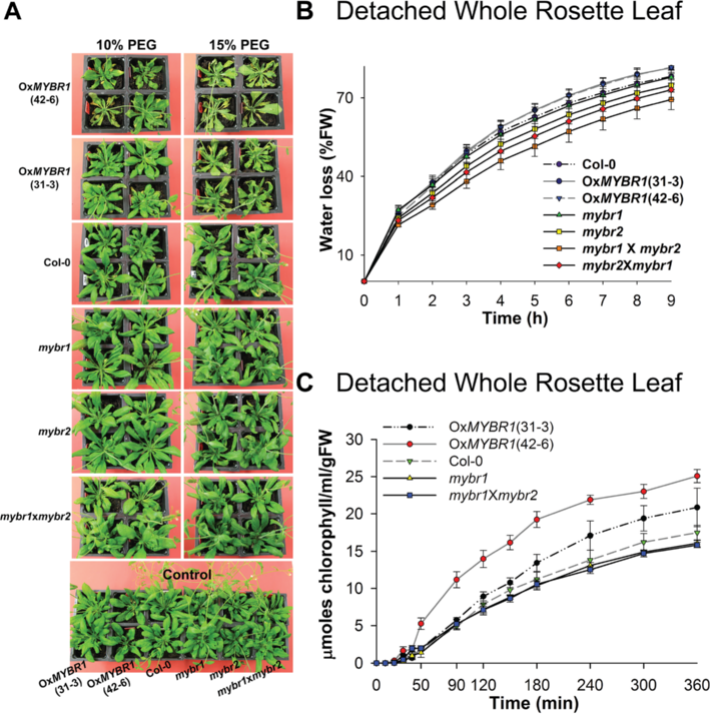
**Gain of At*MYBR1 *function results in reduced drought tolerance. (A)** Reduced drought tolerance in the Ox*MYBR1* (35S_pro_:*MYBR1*) plants -- lines 31–3 and 42–6. PEG at 10% and 15% concentrations was applied to 35 d old plants of Ox*MYBR1*, *mybr1*, *mybr2* and *mybr1*x*mybr2* and WT (Col-0). Eight plants were used for each treatment. Pictures were taken 5 d after PEG treatment. **(B)** Detached whole rosette leaf water-loss from 20 d old plants. Transpirational water loss and standard error was calculated at each time point (bar; n = 6). The P-value of two factor ANOVA is 4.7E-18. **(C)** Rate of chlorophyll leakage from detached whole rosette leaf; standard error (bar; n = 6).

To define the function of *MYBR1* during drought stress, plants were treated with 10% and 15% polyethylene glycol (PEG) for 5 d. Consistent with the down regulation of the stress responsive genes (Figure 2), Ox*MYBR1* plants showed the highest degree of stress (leaf curling, bleaching) following PEG treatment and homozygous *mybr1*x*mybr2* plants showed the least damage (Figure 3A). Subsequently, we found that Ox*MYBR1* rosettes lost water and chlorophyll faster than WT and loss-of-function mutants (Figure 3B and 3C). Therefore, consistent with the down regulation of stress responsive genes, the data suggests that Ox*MYBR1* plants transpired water faster and are consequently less drought tolerant than WT plants.

Drought stress was also imposed by withholding water for 18 d on seedlings from all available genotypes. Surprisingly, we recorded a 100% survival in all 10 replicates of Ox*MYBR1* (42–6) (Additional file 1: Figures S2A and S2B). Results obtained when drought stress was imposed by withholding water were opposite to what was observed following PEG mediated drought stress.

When performing the soil drying experiments above it was observed that, when plants from each genotype were provided with equal volumes of water, the Ox*MYBR1* soil dried more slowly than other genotypes. Therefore to investigate the contradictory results obtained by PEG and soil drying experiments, we conducted transpirational water loss assays on whole plants (Additional file 1: Figure S2C). In these experiments, soil water loss by evaporation was prevented so that plant water use could be monitored. The results showed that Ox*MYBR1* plants lost water slower than WT and mutants, which is contrary to what was observed in detached whole rosette leaves (Figure 3B). In other words, Ox*MYBR1* plants extracted and/or used less water from the soil than other genotypes, even though the transpirational capacity of the detached leaves was relatively higher. Therefore, we investigated the water conservation characteristic of Ox*MYBR1* further. We measured the soil water content after imposing an 8 d drought. Results confirmed that water uptake was less in Ox*MYBR1* than in WT and loss-of-function mutants during drought (Additional file 1: Figure S2D). Under normal (unstressed) growth conditions, Ox*MYBR1* plants grew more slowly than WT and loss-of-function mutants (Additional file 1: Figure S2A) and we show later that OxMYBR1 lines have shorter primary roots than other genotypes. Therefore, with respect to *MYBR1* function, we conclude that reduced water use in whole Ox*MYBR1* plants in drying soil is not due to a genetically determined reduction in transpiration but is rather a consequence of lower biomass leading to slower depletion of soil moisture. The reduced growth rate of Ox*MYBR1* lines was also noted by Jung et al. [15] and in soybean by Seo et al. [25].

As an aside, we note that the residual water content of *mybr2* material was slightly higher than the other loss-of-function lines (Additional file 1: Figure S2D). This may be an indication that the functions of *MYBR2* are not identical with those of *MYBR1*, as discussed later.

### MYBR1_pro_:GUS is expressed under abiotic stress and during senescence, mechanical wounding and floral organ abscission

**Figure 4:**
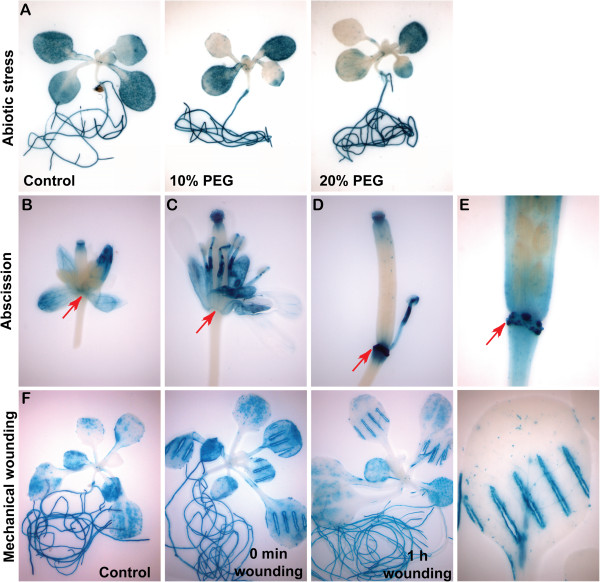
**At*MYBR1 *promoter drives *GUS *expression during abiotic stress, floral organ abscission and mechanical wounding.** Histochemical localization of GUS activity was performed by staining with X-gluc for different time intervals as described below and in Methods. Panel **(A)** Significant reduction of *GUS* expression in leaves after drought stress. However, intense *GUS* expression in roots was similar to control. **(B)** A closed flower was opened manually by forceps before *GUS* staining was performed. **(C)** A fully opened flower. **(B)** and **(C)** No *GUS* expression was found on pedicel at the point where the sepal, petal and anther join (shown by arrows). **(D)** and **(E)** In contrast, intense *GUS* expression was observed at pedicel connecting sites of floral organs after abscission (arrowheads). **(E)** A silique fully abscised with floral organs, was stained briefly (2 h) for GUS. Magnification of the connections between floral organs and pedicel shows *GUS* expression only at attachment sites on pedicel. Panel **(F)** Leaves were wounded with hemostats. Intense *MYBR1* promoter driven *GUS* expression was observed around the wound relative to control.

A 2.7 kb promoter fragment of *MYBR1* including the 5′UTR was fused to the *β-GLUCURONIDASE* (*GUS*) reporter gene (*MYBR1*_pro_:*GUS)* and the expression of *MYBR1* was examined histochemically. GUS staining was performed on homozygous T_2_ and T_3_ plants. In 13 d old seedlings (Additional file 1: Figure S3A), *GUS* expression driven by the *MYBR1* promoter was observed in cotyledons and true leaves. In contrast to very high *GUS* expression in cotyledons, *GUS* expression was lower in younger true leaves relative to older leaves and was absent in the newly emerged leaves. Intriguingly, *GUS* expression was observed in patches in younger leaves and was absent around the vascular regions of both older and younger leaves hinting that *MYBR1* could be involved in senescence since this pattern was reminiscent of the development of visible senescence in leaves reviewed in [9]. Under normal conditions, expression of *GUS* was also observed in hydathodes of all leaf margins as well as embryo, suspensor, endosperm, root, stigma, sepal, petal and anther filament but was absent in stem, cauline leaf, anther, silique and testa (Figure 4A, C, D and E and Additional file 1: Figure S3). *GUS* expression was observed in embryo and endosperm dissected from siliques at developmental stages from 6–18 DPA as well as from dry and imbibed (30 min – 99 h) mature seeds (Additional file 1: Figure S3B and S3C). The intensity of GUS staining increased with development in embryos but remained constant in endosperm except at 6 DPA when the *GUS* expression was lower. *GUS* expression was high and remained constant in embryos collected from dry seeds and seeds imbibed up to 24 h but declined subsequently. *GUS* expression in endosperm of dry and imbibed seeds remained high.

Water stress significantly reduced *GUS* expression driven by the *MYBR1* promoter in leaves but not in roots relative to control (Panel A in Figure 4). Drought induced reduction of *MYBR1* expression is consistent with the reduced expression of *MYBR*1 under drought treatment observed by Huang et al. [8].

*GUS* reporter activity was rapidly and strongly induced immediately after mechanical wounding of leaves (Panel F in Figure 4). Similar high *MYBR1* expression was also observed at the abscission zone (AZ) on the pedicel following sepal, petal, and anther filament abscission (Figure 4D and 4E). Prior to the abscission of floral organs (Figure 4B and 4C), no *GUS* expression was visible on the pedicel. However, we did not observe either accelerated- or delayed/abolished floral organ shedding in *MYBR1* loss-and gain-of-function mutants, suggesting that the high *GUS* expression at the AZ is due to the wounding response.

### AtMYBR1 delays leaf senescence

**Figure 5:**
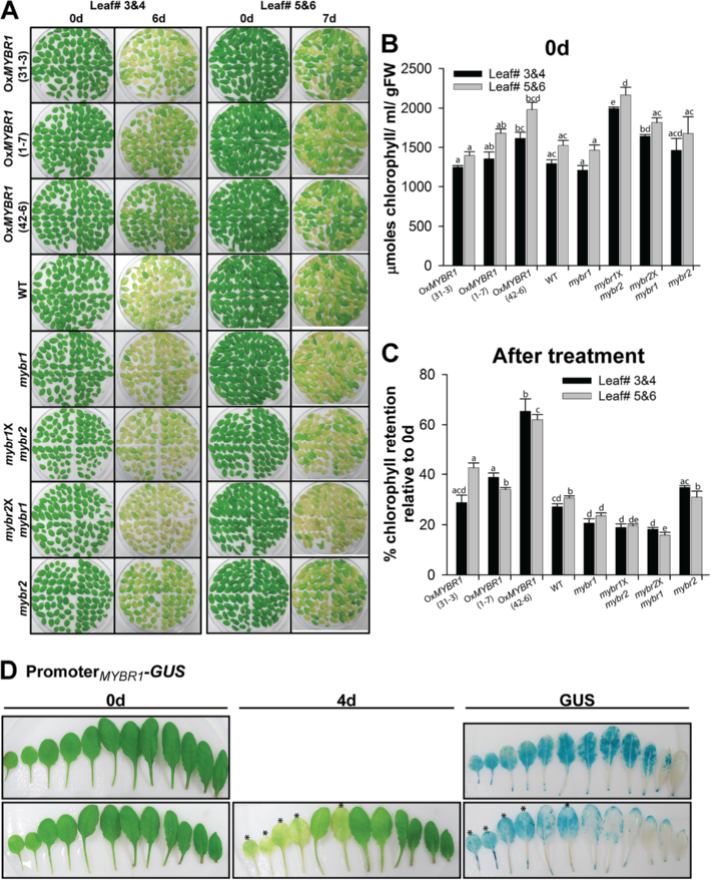
**Effect of *MYBR1 *on leaf senescence in a detached leaf assay. (A)** True leaves numbers 3–6 were harvested from 30 d old soil grown plants and incubated on filter paper wetted with 3 mM MES buffer (pH 5.7). Leaves 3 and 4 were photographed after 6 d treatment and leaves 5 and 6 were photographed after 7 d treatment. Leaves from Ox*MYBR1* plants of three independent lines (#31-3, 1–7 and 42–6) showed delayed senescence relative to other genotypes and leaves from *mybr1* and double mutant plants exhibited premature leaf senescence relative to leaves from WT (leaves 5 and 6) and Ox*MYBR1* plants (all leaves). Two sets of experiment were carried out as above (A) in four replicates and 12 plants in each replicate. Statistical significance was determined using one-way ANOVA with Tukey using the statistical software ‘R’ (P < 0.05). **(B)** In one set of experiments, chlorophyll was extracted and measured on 0 d. The chlorophyll content was higher in one line of Ox*MYBR1* (#42-6) and reciprocal double mutants of *mybr1* and *mybr2* than other genotypes. **(C)** In another set, chlorophyll was extracted and measured on 6 d for leaves 3–4 and on 7d for leaves 5–6 and the percentage chlorophyll retention was calculated relative to 0 d from (B). Chlorophyll retention was generally higher in Ox*MYBR1* genotypes. **(D)** Using three independent homozygous *MYBR1*_pro_:GUS lines (#5-1, 7–6 and X1-4), experiments were carried out as above (A) in two replicates. All leaves from each plant were harvested. GUS staining was performed on 0 d (untreated) and after 4 d of dark induced senescence. Asterisks indicate yellow leaves before and after GUS staining. After the treatment, GUS staining was higher in senescent leaves, but in green leaves was lower than corresponding control leaves.

Early in leaf senescence chloroplasts disassemble with subsequent degradation of chlorophyll and visible leaf yellowing. To further investigate the role of *MYBR1* in leaf longevity, detached rosette true leaves numbers 3–6 (counted by order of emergence), from 30 d old soil grown plants were incubated in buffer as described [26] in two different sets. Leaves were photographed and the chlorophyll content was quantified on 0 d for one set and after 6–7 d of dark-induced senescence treatment for the other set. In freshly harvested leaves, the chlorophyll content was higher in one line of Ox*MYBR1* (#42-6) and two reciprocal double mutants of *mybr1* and *mybr2* than the rest of the genotypes (Figure 5B). Following dark-induced senescence, Ox*MYBR1* lines showed increased leaf longevity and slowed chlorophyll degradation relative to WT leaves (Figure 5A and 5C). Interestingly, increased leaf longevity in Ox*MYBR1* lines was in contrast to that in *mybr1* and reciprocal *mybr1 & mybr2* mutants which exhibited early leaf senescence and accelerated chlorophyll degradation relative to WT (Figure 5A and 5C) although the chlorophyll content before treatment was relatively high in *mybr1* and *mybr2* double mutants (Figure 5B). Indeed, among the eight genotypes, leaves of *mybr1 & mybr2* were least green following senescence treatment and showed the fastest chlorophyll breakdown (Figure 5A and 5C).

To further investigate the role of *MYBR1* in leaf senescence, excised leaves of 37 d old transgenic *MYBR1*_pro_:*GUS* were stained for GUS before and after dark induced senescence (Figure 5D). In untreated plants, GUS staining was strong in older leaves and was absent in younger leaves and in vascular region. GUS staining became weaker overall in dark-treated leaves relative to fresh leaves but among the dark treated leaves, there was more GUS staining in senescent, yellow leaves (indicated by asterisks) than green ones, further suggesting that *MYBR1* plays a role during leaf senescence.

### AtMYBR1 regulates the expression of senescence genes

To investigate whether *MYBR1* regulates the expression of senescence genes, we compared the differentially expressed gene lists of Table 2 with microarray data obtained by van der Graaff et al. [22] on various stages and types of leaf senescence (NS: developmental senescence; DIS: darkening-induced senescence; DET: senescence in dark-induced detached leaf). The number of common genes between the two microarray analyses is 852 (Additional file 3: Table S4) which covers 52% of our differential gene list. The increase of *MYBR1* induction with the progression of senescence is high in NS, low in DIS and DET whereas *MYBR2* induction is slightly increased in sink-to-source transition (5 week) stage of NS [22].

The regulation of these 852 common genes by ABA, *MYBR1* and senescence revealed the interaction between ABA and *MYBR1* during senescence (Additional file 1: Table S5 and Additional file 3: Table S4 online). Out of 165 genes in class A (Table 2, repression of ABA response), 88% were induced by both ABA and NS but repressed by *MYBR1,* clearly demonstrating that *MYBR1* is a suppressor and ABA is an activator of leaf senescence. Furthermore, out of 261 genes in class C (MYBR1 independent ABA repression), 95-96% genes are downregulated by NS, DIS and DET and from 146 genes in class G (MYBR1 independent ABA activation), 86% are upregulated by NS in the same direction as by ABA showing a significant role of ABA in leaf senescence. Of a large number of common genes (161; class B), 89-93% are downregulated by ABA, *MYBR1* and senescence and many of these genes are involved in protein synthesis (23 ribosomal protein genes), photosynthesis (31 genes), auxin responses (6 genes) and biotic stress (5 genes).

**Figure 6:**
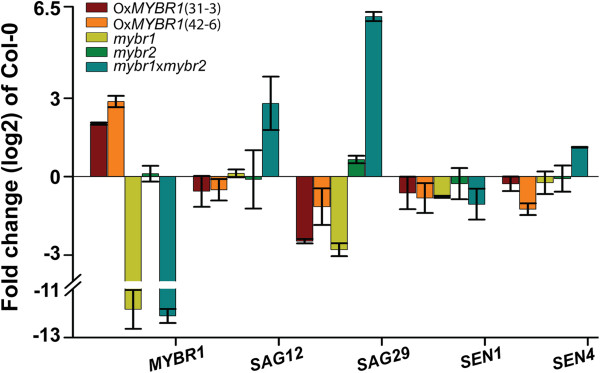
***MYBR1 *and *MYBR2 *regulate expression of some senescence-related genes.** QRT-PCR was performed on total RNA extracted from rosette leaves numbers 3–5 of 21 d old soil grown plants of WT (Col-0), gain- and loss-of *MYBR1* function as well as *mybr2* and double mutant *mybr1*x*mybr2*. Standard error (n = 2) of biological repeats are indicated.

Furthermore, we performed QRT-PCR on senescence marker genes in rosette leaves numbers 3 and 4 in all the genotypes (primers for QRT-PCR are listed in Additional file 1: Table S3). Consistent with our detached leaf senescence analysis (Figure 5), accelerated leaf senescence of *mybr1*x*mybr2* was associated with upregulated expression of *SAG12*, *SAG29* and *SENESCENCE4* (*SEN4*) relative to all other genotypes (Figure 6). *SAG29* expression was 70 (±8.5) fold higher in *mybr1*x*mybr2* relative to WT. The expression of *SAG12*, *SAG29*, *SEN1* and *SEN4* were downregulated in Ox*MYBR1* relative to WT, consistent with the senescence-suppressing effect of *MYBR1*. Changes in expression of these genes in the single mutants *mybr1* and *mybr2* were generally small. However, expression of *SAG21* and *SEN1* did not show a reciprocal relationship between gain and loss of function genotypes. Nevertheless, it is apparent that *MYBR1* negatively regulates senescence based on 3 of the 4 marker genes.

Protein degradation occurs during senescence via different pathways such as autophagy (APG) and the 26S proteosome and components of both pathways were transcriptionally activated during senescence [22,27]. Our differential gene list contains 87 genes involved in protein degradation pathways (Additional file 3: Table S4). Here we report the repression of 44 genes by Ox*MYBR1,* where 31 genes encode components of E2 (ubiquitin-conjugating protein (UBC)), E3 ubiquitin-ligase complex and 20S core particle of 26S proteosome and three genes are *APG8a*/8*f*/*8e*. Interestingly, only three genes in the ubiquitin pathway - *PHD* finger family, *EARLY-RESPONSIVE TO DEHYDRATION 16/UBIQUITIN ETENSION PROTEIN 1* (*ERD16*/*UBQ1*) and *SUPPRESOR OF NIM1-11* (*SON1*) were upregulated by Ox*MYBR1. SON1* is an F-box protein component of E3-ubiquitin ligase complex which negatively regulates, through the ubiquitin-proteosome pathway, a novel defense response that is independent of systemic acquired resistance [28]. On the other hand, 45 genes involved in protein degradation were activated by ABA in both WT and *mybr1,* and many of them are also upregulated by NS, DIS and DET [22].

### Content of endogenous cytokinins and jasmonic acid

**Figure 7:**
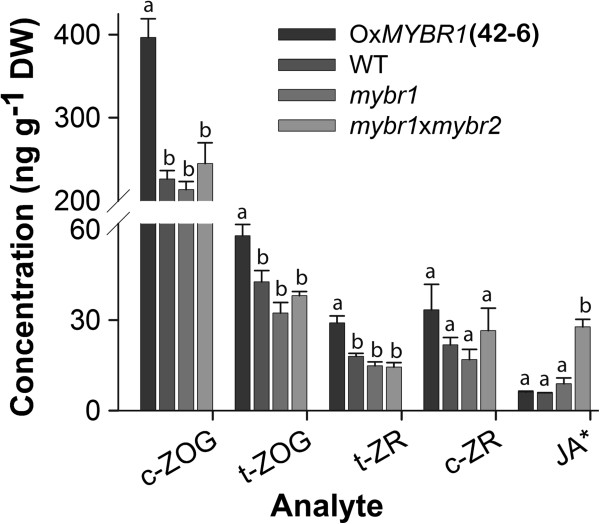
**Endogenous levels of CKs and JA in rosette leaves numbers 3–5 of 25 d old soil grown plants of WT, gain- and loss-of *MYBR1 *function and double mutant *mybr1*x*mybr2*.** Leaves were harvested from at least 8 plants per replicate per compound measured. Standard error was calculated from each hormone (n = 4). CKs measured include: c-ZOG, *cis*-zeatin-O-glucoside; t-ZOG, *trans*-zeatin-O-glucoside; t-ZR, *trans*-zeatin riboside; c-ZR, *cis*-zeatin riboside. JA*: Concentration was measured as ng g^-1^ FW.

To investigate the role of *MYBR1* in relation to hormonal pathways, endogenous hormone levels were measured quantitatively by LC-MS/MS in rosette leaves numbers 3 and 4 of three weeks old plants (Figure 7). *trans*-Zeatin (t-Z) and N^6^-(Δ^2^-isopentenyl) adenine (iP) and their sugar derivatives are the major cytokinins (CKs) in *Arabidopsis*[29]. Levels of several CKs were significantly increased in Ox*MYBR1* relative to other genotypes. On the other hand, JA was significantly higher in *mybr1* x *mybr2* relative to other genotypes. This suggests that suppression of leaf senescence by *MYBR1* is associated with increases in CKs and conversely that increased senescence is associated with higher JA.

In our microarray data, Ox*MYBR1* (comparison: Ox*MYBR1* treated with PBI425 versus untreated Ox*MYR1*) downregulated *ARR4* (Table 2), a transcriptional repressor of CK signaling [30]. However, the down regulation of *ARR4* by ABA (Table 2) and senescence [22] is contradictory and may be due to a feedback effect. Furthermore, we did not detect differential expression of genes involved in CK metabolism but a posttranscriptional regulatory effect of *MYBR1* on expression of these genes cannot be ruled out.

Ox*MYBR1* leaves contained similar levels of ABA and its metabolite dihydrophaseic acid to those measured in WT/*mybr1* and *mybr1*x*mybr2*. Levels of SA and IAA were also not significantly altered. It is surprising that ABA levels remained constant despite the strong effect of At*MYBR1* overexpression on ABA responses. To investigate further, we performed qRT-PCR (primers for QRT-PCR are listed in Additional file 1: Table S3) on six ABA downstream effectors; *ABI5*, *EEL* and *ABF1/2/3/4* in plants of Ox*MYBR1* (lines 42–6 and 31–3), WT, *mybr1/2* and *mybr1* x *mybr2* (Additional file 1: Table S3). No significant differences of expression of these genes were evident among the genotypes tested.

### MYBR1 mis-expression affects leaf and root morphology

**Figure 8:**
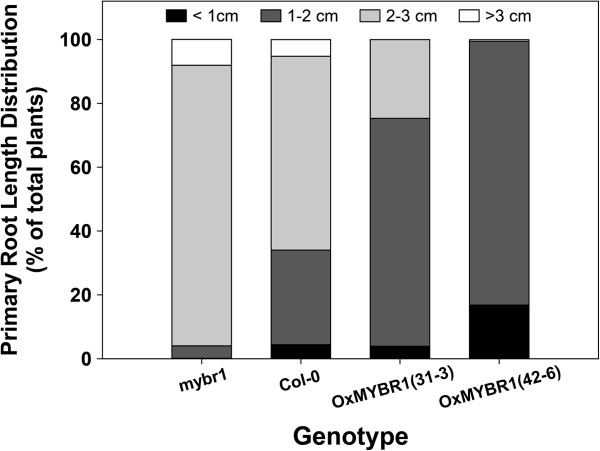
**Primary root lengths of *MYBR1 *genotypes.** Primary roots were measured in soil-grown 8 day old plants. The results are expressed as the percentage of roots of each genotype that fell within the four indicated length ranges. For each genotype, the total number of measured roots and the total range of lengths in cm were: Ox*MYBR1* 42–6, 380 (range 0.54-2.12); Ox*MYBR1* 31–3, 203 (range 0.71-2.71); Wild type, 270 (range 0.62-3.57); *mybr1*, 372 (range 1.22-3.59).

We examined the roots of gain-and loss of *MYBR1* function genotypes (Figure 8). Primary roots of Ox*MYBR1* lines were drastically shorter, whereas those of *mybr1* were notably longer relative to WT. This shorter root phenotype of Ox*MYBR1* may contribute toward reduced water uptake in the Ox*MYBR1* lines as noted earlier, and may help explain the differences between results of PEG treatments and soil drying experiments described above.

**Figure 9:**
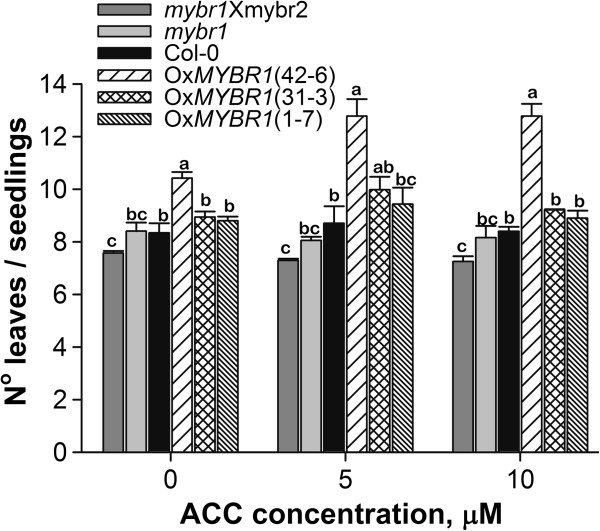
**Leaf number in *MYBR1 *genotypes.** Seeds from WT, *mybr1*, *mybr1*x*mybr2* and three lines of Ox*MYBR1* (42–6, 31–3 and 1–7) were germinated on MS plates and 8 d old seedlings were transferred to fresh plates without and with 5 and 10 μM ACC. Leaf number was counted 17 d after transfer. Control and ACC treated experiments were conducted in four replicates and each replicate contained four seedlings per genotype. The experiment was repeated three times. Standard error was calculated at each time point (n = 12). Two-way ANOVA resulted in significant differences between genotypes but no significant difference between control and ACC treatments. There were increased numbers of rosette leaves in OxMYBR1 lines relative to other genotypes (especially line 42–6) and a significantly reduced number of leaves in the *mybr1* and *mybr2* double mutant relative to most other genotypes with and without ACC. However, ACC treatment qualitatively enhanced differences between genotypes.

The *Arabidopsis* mutant *amp1,* with a high level of endogenous CKs, had increased numbers of rosette leaves [31]. Conversely, plants overexpressing catabolic CK oxidases had fewer leaves than WT plants [32]. We counted rosette leaves in seedlings of two lines of Ox*MYBR1*, WT and *mybr1*. Seedlings of Ox*MYBR1* lines had consistently more rosette leaves relative to other genotypes and double mutant *mybr1*x*mybr2* had fewer leaves relative to all other genotypes (Figure 9). Differences between genotypes are slightly enhanced by ACC treatment. Using two way ANOVA, there is a consistent, statistically significant difference in leaf number between *mybr1* x *mybr2* and the Ox*MYBR1* lines but no significant difference between control and ACC treatments.

### MYBR1 physically interacts with PYL8 and INO

Further information on the mechanistic role of MYBR1 in signaling was obtained by identifying protein-protein interactions using the yeast 2-hybrid system. Initially PYR1-LIKE8 (PYL8) and INNER NO OUTER (INO) proteins were identified as interacting with MYBR1 by screening an *Arabidopsis* cDNA library made from different stages of vegetative and floral tissues with a full length *MYBR1* fused to the DNA-binding (BD) domain of the yeast GAL4 protein. It has been shown by many groups that the 14 members of the PYR/PYL/RCAR family are intracellular ABA receptors that interact with and inhibit several PP2C-type protein phosphatases including ABI1, ABI2, HAB1 and PP2CA [2-4]. *INO* encodes a YABBY-type TF and is required for both polarity determination and outer integument initiation in ovule development [33].

**Figure 10:**
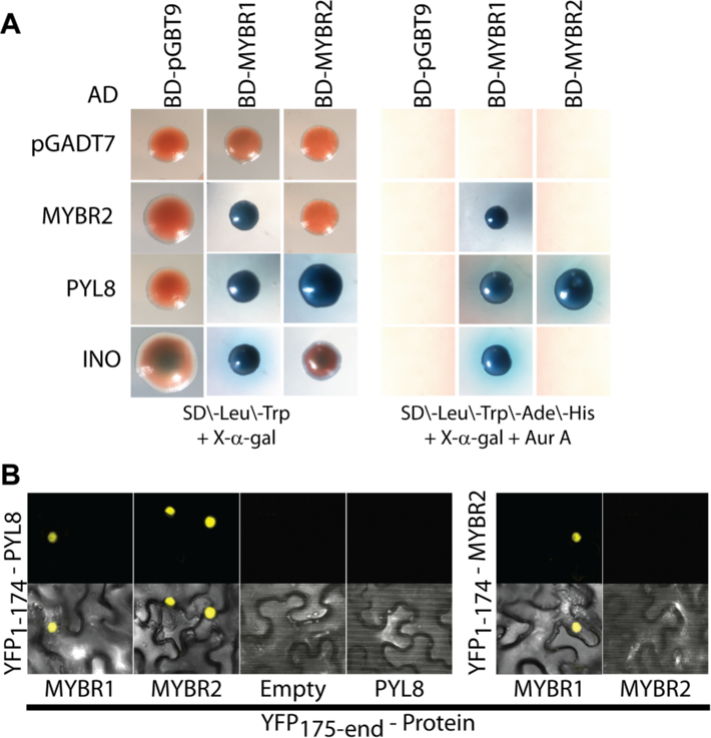
**Physical interaction of MYBR1 and MYBR2 with PYL8, MYBR2 and INO as determined by the yeast two-hybrid method and BiFC. (A)** Blue color from galactosidase activity indicates interaction between proteins produced from the bait (top row) and prey (vertical axis) vectors. **(B)** BiFC of the interactions of PYL8 with MYBR1 and MYBR2 and of MYBR2 with MYBR1in *N. benthamiana* leaf epidermal cells. Top panel shows the signal from eYFP, reconstituted from YFP1-174 aa -PYL8 and YFP175-end aa -MYBR1 & MYBR2 and from YFP1-174 aa -MYBR2 and YFP175-end aa -MYBR1. Bottom panel presents eYFP and transmitted light detector signals.

MYBR1 interaction with PYL8 encouraged us to subsequently fuse all 14 *PYR/PYL/RCAR* genes as well as *INO* and *MYBR2* to the transcription-activation (AD) domain of the yeast GAL4 protein. Interestingly, only PYL8, INO and MYBR2 interacted with MYBR1 (Figure 10). Interaction of MYBR1 and PYL8 was confirmed by three independent experiments using a more stringent screening of positive clones on four drop out media (SD/-Leu/-Trp/-His/-Ade) in the presence of antibiotic Aureobasidin A. Furthermore, we also fused the full length *MYBR2* to the BD-domain and found that it also interacts only with PYL8 out of the 14 PYL family members. But MYBR2 showed no interaction with INO which suggests that, despite their shared roles in stress response and senescence, MYBR1 and MYBR2 have some non-redundant functions. In Figure 10, yeast colonies resulting from interaction of AD-pGADT7, -MYBR2, -PYL8, and -INO with BD-MYBR1 were smaller compared to those with BD-MYBR2 and BD-pGBT9. It should be noted that we observed slight autoactivation and notable toxicity/reduced cell growth from high expression of MYBR1 using the pGBKT7 plasmid and hence used the lower expressing plasmid pGBT9.

Next, we examined whether the protein-protein interactions described above are affected by phytohormones (Additional file 1: Figure S4). In addition, MYBR2 is reported to modulate auxin signaling [21] and therefore we also tested inhibitors of auxin signaling (PCIB) and transport (TIBA and NPA). However, the above interactions were constitutive and not affected by phytohormone additives.

We performed bimolecular fluorescence complementation (BiFC) assays in *Nicotiana benthamiana* leaf epidermal cells to independently verify the interactions of PYL8 with MYBR1 and MYBR2 and of MYBR1 with MYBR2. It has been shown previously that PYL8 and MYBR1 are localized in the nucleus [15,34] and interaction between MYBR2 and ARF7 occurs in the nucleus [21]. All interactions of PYL8 with MYBR1 and MYBR2 and of MYBR1 with MYBR2 are high with consistent fluorescent signal (Figure 9). Yeast two-hybrid and BiFC approaches confirmed the interactions of PYL8 with MYBR1 and MYBR2 and between MYBR1 and MYBR2 and showed that PYL8 may modulate the binding of MYBR1 and MYBR2 to DNA and/or that both MYBR1 and MYBR2 may modulate PYL8 function. The interaction of MYBR1, 2 with only PYL8 but not with other members of PYR/PYL/RCAR family suggests that these interactions define very specific functional roles.

## Discussion

We previously identified *MYBR1* as a weakly ABA responsive gene [14] and here we provide evidence that it is a repressor of ABA signaling during seedling growth, drought and senescence. It is now clear that *MYBR1* is part of the ABA/abiotic stress response and wounding/ abscission response networks, both of which involve senescence responses. *MYBR1* acts as a negative regulator (feedback repressor) of responses to stress, wounding and abscission in favor of normal growth and development. *MYBR1* is by no means unique in its ability to negatively regulate ABA and stress responses. Other examples include the AP2 domain TFs *ABA REPRESSOR1* (*ABR1*) [35] and *ETHYLENE RESPONSE FACTOR7* (*ERF7*) [36] and the homeodomain protein *HB6*[37].

**Figure 11:**
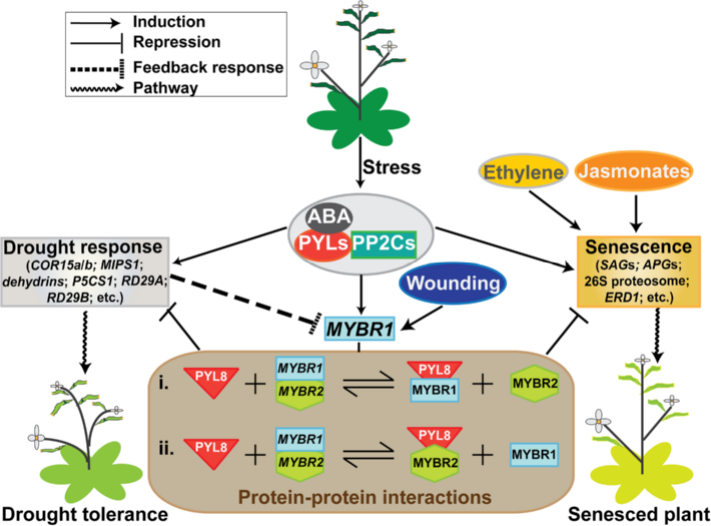
Model of MYBR1 mechanism of action and effects.

Our original observations that *MYBR1* was induced by PBI425, induced weakly by ABA, repressed by drought and paradoxically induced by rewatering after drought stress [8,14] have been confirmed and can now be rationalized. Under non-stressed conditions, ABA treatment produces unnecessary stress responses and *MYBR1* induction blocks these responses to restore normal patterns of gene expression. Under water stress conditions *MYBR1* is not expressed, allowing the full effects of ABA to be manifested and allows adaptive responses to be maintained during drought stress. On recovery from stress, *MYBR1* expression leads to repression of ABA responses that are no longer required. The regulation and effects of *MYBR1* are summarized in Figure 11.

Although induced senescence during prolonged drought has survival value by conserving water and nutrients [11], there are clearly finely balanced advantages and disadvantages to irreversible loss of vegetative matter. In fact, by introducing a novel feedback mechanism to suppress drought induced senescence in tobacco, Rivero et al. [10] demonstrated striking beneficial effects, suggesting that, in a crop plant context, induced senescence can be disadvantageous. Therefore, it seems that *MYBR1* is a component of an endogenous homoeostatic mechanism to balance growth, high seed production and risk of death versus senescence, survival and minimal seed production. Given that senescence of older leaves is a normal stage of leaf development, *MYBR1* appears to also play a role in determining the normal length of the leaf adult phase.

Senescence induces protein degradation pathways [22,27,38] and the effects of *MYBR1* are associated with reduced/delayed expression of ubiquitin- and autophagy mediated protein degradation and increased production of CKs. Previous studies have associated drought-induced leaf senescence with reduced CKs [11] and increased CK biosynthesis blocks leaf senescence [39]. Higher levels of CKs, reduced primary root growth and more adult leaves in Ox*MYBR1* lines are also consistent with increased CK effects. However, there are other hormonal interactions. *MYBR1* appears to repress jasmonate effects – which likely also contributes to suppression of wounding responses. Jung et al. [40] demonstrated that *MYBR1* was induced by jasmonate and also showed that jasmonate responses were repressed. More recently Shim et al. [41] show that *MYBR1* represses JA defense responses and activates salicylic acid-mediated defenses via WRK70 leading to enhanced responses to biotrophic pathogens and attenuated responses to necrotrophic pathogens.

We propose a model of *MYBR1*-repression of ABA signaling during drought and senescence (Figure 11). It has been shown previously that PYL8 is localized in both cytoplasm and nucleus and the interaction between PP2C1 and PYL8 takes place in the nucleus [34]. In addition, MYBR1 is also localized in the nucleus [15]. Therefore, the interaction of MYBR1 with PYL8 suggests a direct role of MYBR1 in modulating ABA perception. The uniqueness of the interaction with PYL8 (and with no other PYL) provides an example of receptor specificity - an ABA receptor mediating a specific sub-network of responses. The existence of such effects was suggested by comparison of the effects of ABA analogs in Huang et al. [14]. Previous papers have noted that binding of PYL8 to PP2Cs does not appear to be dependent on ABA, so the regulatory significance of the PYL8-ABA complex is not clear. Increased drought tolerance and ABA hypersensitivity in seed of 35S_pro_:*PYL8* lines showed that PYL8 is an overall positive regulator of ABA signaling [34]. Binding of MYBR1 to PYL8 may block interaction with and inhibition of PP2Cs. Alternatively, PYL8 may regulate MYBR1 binding to DNA. Since PYL8-PP2C binding is independent of ABA, PYL8 may be responsible for constitutive ABA signaling that is independent of ABA itself or ABA may be required to fully potentiate PYL8-PP2C interaction. Future studies will further explore the MYBR1-PYL8 interaction in relation to *MYBR1* function.

The weak phenotypes of the *mybr1* and *mybr2* mutants and the enhanced effects in the double *mybr1 x mybr2* mutant strongly suggest that *MYBR1* and *MYBR2* are partially redundant and the yeast two hybrid data indicates that they may form heterodimers (Figure 11). However, *MYBR2* has mainly been associated with auxin signaling and root development [21], shows differing MYBR2_PRO_::GUS expression patterns compared to MYBR1_PRO_::GUS [21], and has not been distinctly associated with ABA or jasmonate response as our data and others suggest for *MYBR1*[14,15,17,40].The specific interaction of MYBR1 (and not MYBR2) with INO suggests that there are at least some unique functions of MYBR1 not shared by MYBR2. However, the significance of the MYBR1-INO interaction is unknown at this time. *INO* encodes a YABBY-type transcription factor and is only known to be involved in ovule development [33] and there is no specific *MYBR1* phenotype associated with flowers.

The effects of *MYBR1* overexpression in *Arabidopsis* were also studied by Jung et al. [15], but some of their results were significantly different to those reported here. Jung et al. [15] reported downregulation of stress genes but increased stress tolerance and reduced water loss from detached shoots in over-expression lines and obtained similar results in soybean transgenics [25]. Similarly, Persak and Pitzschke [17] reported delayed mortality of an OxMYBR1 line relative to wild type when exposed to toxic levels of salt. For this reason, we focused carefully on identifying the most appropriate approach to measuring drought and water loss. We believe that our results demonstrate that the reduced size of Ox*MYBR1* lines – due to slower growth of above-ground tissues and shorter primary roots – is associated with reduced water use and slower depletion of soil moisture. This phenomenon produced an apparent increase in drought tolerance because the differential size and water use of the *MYBR1* genotypes were not taken into account. To circumvent this issue, PEG treatment (which maintains a specific soil water potential) was used to reveal the increased sensitivity of Ox*MYBR1* lines to water stress (as shown in Figure 3A). Furthermore our microarray results are consistent with reduced stress responses in Ox*MYBR1* lines and careful analysis of microarray results in Table 1 in Jung et al. [15] suggests that many well-known positive effectors or regulators of stress responses (such as *ERD1*, *KIN1*, *COR15a*, *COR15b*, *RAB18*, *RESPONSIVE TO DESSICATION29A* (*RD29A*), *COR47*, *RD29B*, *DELTA1-PYRROLINE-5-CARBOYLATE SYNTHASE1* (*P5CS*), *DREB2A*) were similarly down-regulated in overexpressing At*MYBR1* plants relative to WT plants. However, Jung et al. did not perform experiments that showed the effects of *MYBR1* overexpression on repressing ABA/PBI425-induced genes (as in Figure 1). The differences between our results and Jung et al. [15] in measuring drought tolerance provides a cautionary example of the complexities and subtleties of performing and interpreting drought and water use experiments. Unlike Jung et al. [15] and Persak and Pitzschke [17], we did not investigate salt-stress related phenotypes related to *MYBR1* expression. More recently, Jung et al. [40] suggested that *MYBR1* was induced non-specifically by phytohormones and suppressed jasmonate responses. Our data also suggest an effect of *MYBR1* on repressing JA responses, but show a direct and unambiguous link to ABA signaling as described above.

## Conclusions

In the last few years, considerable information has accumulated on the involvement of *MYBR1* in stress-related MAPK signaling. However, the function of the gene in relation to stress responses has remained unclear. This study reveals that *MYBR1* is a component of ABA signaling and appears to be involved in feedback maintenance of adult, pre-senescent growth, especially under conditions of stress and wounding. As such it provides an example of a transcription factor that integrates, balances and co-ordinates hormonal, developmental and environmental signals.

## Methods

### Plant materials, growth conditions and treatment

*Arabidopsis thaliana* plants were grown under long-day conditions in a growth cabinet at 22°C and 40% humidity with 16 h of 80 μE light and 8 h dark cycles. Seeds were surface sterilized as follows: seeds were washed aseptically; once with 70% ethanol for 30 sec and three times with 20% bleach for 5 min followed by four washes with sterile water. Water was removed after the final wash and 0.2% agar solution was added to facilitate placing seeds on Murashige-Skoog (MS) + 0.8% agar media without sucrose. Seed stratification was performed at 4°C, in the dark for 3 d. Since growth rates differ slightly between genotypes, care was taken that observed differences between genotypes at specific times were consistent and not artifacts of different developmental stages.

For microarray experiments, growth of plants, treatment of 5 week old plants with 20 μM PBI425 for 24 h and above ground tissue collection were done as described in Huang et al. [14].

For root phenotyping of seedlings following seed stratification, agar plates were transferred to a controlled environment cabinet. Eight days after stratification, seedlings were photographed using a digital camera and root lengths were measured using ImageJ software (version 1.37v, NIH, USA).

For generation of *mybr1*x*mybr2* double mutant, T-DNA insertion lines of (*mybr2*) SALK_67655 was obtained from the Arabidopsis Stock Center (http://arabidopsis.org). This loss-of-function mutation in this line is caused by T-DNA insertion into an exon. *mybr2* homozygous plants were identified by PCR as described [42]. Homozygous plants of *mybr1*[14] and *mybr2* were crossed reciprocally. Homozygous double mutants *mybr1*♀ x *mybr2* ♂ and *mybr2*♀ x *mybr1*♂ were identified by PCR [42].

### PEG treatment

Following stratification at 4°C, plants were grown in soil (Sunshine 3 Mix from Sun Gro Horticulture Inc.) for 17 d in a growth chamber at 22°C and 64% humidity with 16 h of 150 μE light and 8 h dark cycles, then transplanted individually into 2″x 2.5″ pots filled with 90 ml sand: soil (2:1) mix. Pots were watered with 30 ml Hoagland solution. We found that maintaining high humidity is crucial in this experiment. Plants were watered as needed and after 20 d, 50 ml of 10% or 15% PEG solutions was added to each pot. After 30 min to allow drainage, pots were transferred to fresh tray holders. Pictures were taken 5 d after PEG treatment.

### Transpirational water loss assays of detached whole rosette leaf and whole plants

Plants were grown as described above. Whole rosette leaves of 20 d old plants were excised, placed in a weighing boat and weighed at intervals for up to 9 h. Samples were kept at 22°C between weighing intervals.

### Chlorophyll assay

Freshly harvested leaves were weighed and chlorophyll was extracted on 0 d (untreated) and after 6–7 d following dark induced senescence. Chlorophyll extraction and quantification were carried out as described by [43]. Leaves or whole rosettes of *Arabidopsis* were harvested and weighed. Chlorophyll was extracted by placing the tissue in 90% ethanol at 65°C for 3 h until all tissues became chlorophyll free. The amount of total chlorophyll was determined by measuring absorbance at 664 and 647 nm [44] with a Microplate Reader (Synergy H1) from Biotek and using the formula: micromoles of chlorophyll per milliliter per gram fresh weight = 7.93(A_664_) + 19.53(A_647_).

### MYBR1_pro_:GUS plasmid construction, treatments and GUS staining

A 2.7 kb fragment, including the 5′UTR, of the *AtMYBR1* promoter was PCR amplified from *Arabidopsis thaliana* (Col-0) WT genomic DNA using the primers 5′-attB1-gtagtgcgtgtggatatatacatgca-3′ and 5′-attB2-tgattttggaatgttttatcaaactttag-3′ and cloned into pDONR221 using a Gateway BP reaction (Invitrogen). Following sequence verification, the *MYBR1* promoter was then cloned into the *GUS* expression vector pMDC162 [45] with an LR reaction (Invitrogen).

For GUS staining in seedling, flower and silique, homozygous T_2_ and T_3_ seedlings were grown for 13 d on MS medium in the presence of 1% sucrose and were stained for GUS activity for 70 min. For drought stress, seedlings were grown for 7 days and drought was imposed by overlaying 10% and 20% PEG on an agar plate for 44 h followed by GUS staining for 1 h. True leaves of control plants were wounded aseptically with hemostats and 30 min GUS staining was performed at 0 h and after 1 h of wounding. Floral tissues were stained for 16 h unless otherwise stated. GUS staining was performed [46] with X-gluc staining reagent ( 0.1 M NaPO_4_ pH7.0, 10 mM Na_2_EDTA, 0.1% Triton X-100, 1.5 mM K_3_Fe(CN)_6_, 0.5 mM K_4_Fe(CN)_6_, and 2.0 mM X-gluc) at 37°C in the dark after three vacuum infiltrations of 1 min each. After staining, chlorophyll was removed completely by successive washes with 50%, 70% and 80% ethanol with gentle agitation and photographs were taken using a Wild M3Z dissecting microscope equipped with a Leica DFC320 camera.

For GUS staining in embryo and endosperm, plants were grown in growth chambers as described above. Siliques were collected at 6, 9, 12, 15 and 18 days post anthesis (DPA) and were fixed in 20% acetone for >24 h at −20°C prior to embryo dissection followed by 30 min GUS staining. Dry and imbibed seeds at different time points were also fixed, dissected and then stained as described above.

### Detached leaf senescence assay

Plants were grown on soil. Rosette true leaves numbers 1–4 as counted by order of emergence (cotyledons were excluded), were excised and incubated with their abaxial sides down on two pieces of 3 MM paper wetted with 10 ml of 3 mM MES (pH 5.7) without or with different concentration of (+)-ABA, 1-aminocyclopropane-1-carboxylic acid (ACC), benzyl amino purine (BP), or MJA at room temperature in the dark [26]. Leaves numbers 1 and 2 were incubated for 5d and juvenile leaves numbers 3 and 4 were incubated for 6–13 d. Leaf pictures were taken after treatment and chlorophyll assay (described above) was performed.

### Quantification of ABA, cytokinins and their metabolites and JA by LC-MS/MS

The plant hormone analysis was performed by high performance liquid chromatography-electrospray tandem mass spectrometry (HPLC-ES-MS/MS) using deuterated internal standards, as described [47,48]. The analysis of free salicylic and jasmonic acid using HPLC-ES-MS/MS with deuterated internal standards will be presented elsewhere (Han et al., unpublished).

### RNA extraction and microarray labeling, hybridization and data acquisition

Total RNA was extracted from frozen tissues of four independent biological replicates as described [49] with a slight modification. Instead of extraction buffer RLT, a mix containing 10 mM Tris–HCl pH 7.5; 0.1 M NaCl; 1 mM EDTA and 1% SDS was used. RNA quantification was performed by measuring optical density at 260 nm. Microarray labeling, hybridization, scanning and data acquisition were done for oligonucleotide microarrays obtained from the University of Arizona according to Huang et al. [14]. However, microarray labeling, hybridization and slide washing for Agilent Technologies Arabidopsis 4x44k arrays (version 4, product# G2519F, design ID 021169) were performed according to the manufacturer’s protocol using low input Quick Amp Labeling Kit for two color (Agilent Technologies; cat# 5190–2306) [50]. In short, 200 ng total RNA was used for cDNA synthesis and 2.5 h for cRNA amplification. Two μg each of cyanine 3- and 5-labeled amplified cRNA was hybridized to each array. After washing, each slide was scanned using Axon 4000B scanner with a resolution of 5 μm/pixel. Data acquisition was done as described above.

### Microarray data analysis

Signal intensity normalization (method: Print-tip loess), filtering bad spots and control spots, filtering minimum channel intensity (intensity for both channel should be <500 in most cases) and correlation coefficient among replicates were performed in BASE [51]. Quality control on sample data was performed in GeneSpring GX 10.0.2 (Agilent). To obtain statistically differentially expressed gene sets, a t-test against zero along with Benjamini-Hotchberg multiple testing correction and with a 0.05 p-value cut-off were performed in GeneSpring. Furthermore, biologically significant differentially expressed gene sets were obtained by using a threshold fold change ≥ 1.5. The spot visualization feature in BASE was employed for an additional quality control for false positives/negatives. Afterward, log2 expression values for each sample type were uploaded into MapMan ImageAnnotator version 3.0.0RC3. Analysis for statistically significant enriched biological pathways, a Wilcoxon rank sum t-test embedded in MapMan was performed with a p-value cut-off of 0.05 and Benjamini Hochberg multiple testing correction [52]. Gene annotation was done based on TAIR database, MapMan and PlantsUBQ (URL http://plantsubq.genomics.purdue.edu).

### Quantitative RT-PCR

Gene-specific primers for QRT-PCR were designed using PerlPrimer v1.1.14 [53]; http://perlprimer.sourceforge.net and are listed in Additional file 1: Table S3. Total RNA was isolated as described above, from rosette leaves 3 and 4 of three week old plants. Complementary DNA (cDNA) was produced using 2 μg total RNA using QuantiTect Reverse Transcription kit from Qiagen (catalog number 205311) according to the manufacturers instruction. Two biological and two technical repeats were performed with null-template control. Arabidopsis *ACTIN2* was used as a normalization control [14]. cDNAs were diluted 10 times in QRT-PCR reactions for all genes (*MYBR1*, *SAG29*, *SEN1* and *SEN4*) except *SAG12* cDNA which was used without dilution. QRT-PCR was performed with SYBR green SuperScript III Platinum Two-Step qRT-PCR Kit (Invitrogen, 11735–032) according to the manufacturer instructions, on a Stratagene Mx3000P real-time PCR thermal cycler.

### Construction of gene fusions for yeast two-hybrid assays

Open reading frames of *MYBR1* and *MYBR2* and 14 genes of *PYR*/*PYL*/*RCAR*s family ABA receptors and the *GAL4* activation domain (AD) and DNA-binding domain (BD) were constructed in the pGADT7 and pGBT9 vectors, respectively (Clontech). The open reading frames (ORF) of *PYL1*/*2*/*3*/5/*6*/*7*/*8*/9/*10*/*11*/*12*/*13* were PCR amplified from cDNA and the ORF of *PYR1* from an ABRC clone (accession number U15941) using PfuUltra II fusion HS DNA polymerase (Agilent; catalog number 600670) and primers are listed in Additional file 1: Table S3. PCR products were gel purified with a gel extraction kit (QIAGEN; catalog number 28704), were cloned into Gateway vector pDONR221 by a Gateway BP reaction (Invitrogen) and were verified by sequencing using M13 forward and reverse primers. ORFs of *PYL4* and *MYBR2* cloned in pENTR223 were obtained from ABRC clones (accession number G12806 and G14459, respectively) and were verified by sequencing using T7 and M13 forward primers. These 15 different ORFs were then cloned in-frame with the *GAL4*AD in pGADT7 by LR reactions (Invitrogen). ORFs of *MYBR1* and *MYBR2* were cloned in-frame with the *GAL4*BD in pGBT9 using In-Fusion Advantage PCR Cloning kit (Clontech; catalog number 639619) as follows: *MYBR1* ORF was PCR amplified from cDNA and *MYBR2* ORF from an ABRC clone G14459 using primers listed in Additional file 1: Table S3. PCR products were gel purified and verified by sequencing using forward 5′-ttttcgttttaaaacctaagagtc-3′ and reverse 5′-tcatcggaagagagtagt-3′ primers. Plasmid pGBT9 was digested to completion with *Eco*RI and *Bam*HI and column purified (QIAGEN; catalog number 28106). In-fusion cloning reactions between ORFs and linearized pGBT9 were performed according to the manufacturer’s instruction.

### Protein-protein interaction analyses

All gene fusions in pGADT7 and in pGBT9 were transformed into the yeast cell lines Y187 and Y2H Gold, respectively and were grown in the presence of 50 μg/μl kanamycin on media SD/Leu and SD/Trp, respectively, according to the manufacturer’s instructions (Clontech; Matchmaker gold yeast two-hybrid system; catalog number 630489). Auto-activation and toxicity of pGBT9-*MYBR1* and pGBT9-*MYBR2* were tested as described by Clontech. For library screening, transformed yeast Y2H Gold with pGBT9-*MYBR1* was used to screen an *Arabidopsis* normalized cDNA library; Mate and Plate (Clontech; catalog number 630487) which was constructed from different stages of vegetative and floral tissues, cloned in pGADT7-RecAB vector and transformed into the yeast Y187. After 24 h mating, library screening was performed on medium SD/-Leu/-Trp/-His/-Ade in the presence of 20 μg/ml x-α-gal (Gold Biotechnology) and 78 ng/ml Aureobasidin A (Clontech) (QDO/X/A) and grown for 4 d at 30°C. Blue yeast colonies were streaked onto fresh QDO/X/A. Following 3 d growth, plasmids were isolated using the Easy Yeast Plasmid Isolation Kit (Clontech) and cDNA inserts were PCR amplified using LD-AD screening primers (Clontech) and verified by sequencing using T7 primer. For individual clone screening, transformed yeast Y2H Gold with pGBT9-*MYBR1*and pGBT9-MYBR2 and transformed yeast Y187 with each PYR/PYL/RCARs/MYBR2-pGADT7 were mated for 1 d at 30°C and screened on media SD/-Leu/-Trp (DDO), DDO/X/A and QDO/X/A as described by Clontech. Bimolecular fluorescence complementation (BiFC), including preparation of constructs, was performed in *N. benthamiana* epidermal cells according to [50].

### Accession numbers

The Arabidopsis Genome Initiative (AGI) locus identifiers for the genes from this article are as follows: *MYBR1*/*MYBR44* (At5g67300), *MYBR2*/*MYBR77* (At3g50060), *PYL8* (At5g53160), *INO* (At1g23420). SALK T-DNA insertion mutant line of *MYBR1* and *MYBR2* are SALK_039074 and SALK_67655, respectively.

## Competing interests

The authors declare that they have no competing interests.

## Authors’ contributions

AJC conceived the project. AJC and MRJ designed the experiments and JAF and DH provided suggestions on experiments. MRJ performed the experiments and analyzed data in the manuscript unless otherwise stated. DH constructed p35Spro:At*MYBR1* plasmid and generated 35Spro:At*MYBR1* T_o_ seeds. JAF constructed p*MYBR1*pro:*GUS* plasmid and generated *MYBR1*pro:*GUS* T_o_ seeds. YL and JAF screened yeast 2-hybrid library. MRJ, YL and JAF verified the interaction of PYL8 with MYBR1. AJC, MRJ, JAF and DH contributed to the interpretation of results. AJC and MRJ wrote and edited the manuscript. All authors read and approved the final manuscript.

## Supplementary Material

Additional file 1: Figure S1 Experimental Designs of Two Color Arabidopsis Microarray Experiments using Above-Ground Tissues of 5 Weeks Old Plants. **Figure S2.** Reduced Water Uptake by Gain of At*MYBR1* Function Leads to Apparent Drought Tolerance in Plants Overexpressing At*MYBR1*. **Figure S3.** Expression of *MYBR1*_pro_:*GUS* in Vegetative Tissues, Embryos and Endosperm at Different Developmental Stages and after Imbibition of Mature Seeds. **Figure S4.** Physical Interaction of MYBR1 and MYBR2 with PYL8, MYBR2 and INO in the Presence of Various Hormones and Inhibitors of Auxin Signaling and Transport. **Table S2.** Enriched TF Sites (http://www.bioinformatics2.wsu.edu/cgi-bin/Athena/cgi/home.pl). **Table S3.** Primers for QRT-PCR and Gene Cloning for Yeast Two-Hybrid Analysis. **Table S5. ***MYBR1* represses genes associated with leaf senescence. **Additional Methods.** Click here for file

Additional file 2: Table S1 Significant Gene List Obtained from T-Test P-Value Cut-Off ≤0.05 and Fold Change ≥1.5 (Excel File). Click here for file

Additional file 3: Table S4 *MYBR1* Represses Genes Induced by Natural Leaf Senescence (Excel File). Click here for file
