# Supplementary material for: Multiple roles of the transcription factor AtMYBR1/AtMYB44 in ABA signaling, stress responses, and leaf senescence
Source: BMC Plant Biol. 2013 Nov 28;13:192. doi: 10.1186/1471-2229-13-192 (PMC4219380; doi:10.1186/1471-2229-13-192)
Supplement: Additional file 1: Figure S1 — Experimental Designs of Two Color Arabidopsis Microarray Experiments using Above-Ground Tissues of 5 Weeks Old Plants. Figure S2. Reduced Water Uptake by Gain of AtMYBR1 Function Leads to Apparent Drought Tolerance in Plants Overexpressing AtMYBR1. Figure S3. Expression of MYBR1pro:GUS in Vegetative Tissues, Embryos and Endosperm at Different Developmental Stages and after Imbibition of Mature Seeds. Figure S4. Physical Interaction of MYBR1 and MYBR2 with PYL8, MYBR2 and INO in the Presence of Various Hormones and Inhibitors of Auxin Signaling and Transport. Table S2. Enriched TF Sites (http://www.bioinformatics2.wsu.edu/cgi-bin/Athena/cgi/home.pl). Table S3. Primers for QRT-PCR and Gene Cloning for Yeast Two-Hybrid Analysis. Table S5. MYBR1 represses genes associated with leaf senescence. Additional Methods. [file 1471-2229-13-192-S1.pdf]

Supporting Data Online for

Multiple Roles of the Transcription Factor At*MYBR1*/At*MYBR44* in ABA Signaling, Stress Responses, and Leaf Senescence

Masrur R. Jaradat[1], J. Allan Feurtado[1], Daiqing Huang[1], Yongquan Lu[2], Adrian J. Cutler[1*]
[1]Plant Biotechnology Institute, National Research Council of Canada, 110 Gymnasium Place, Saskatoon S7N 0W9, Canada, and
[2]Zhejiang Agriculture and Forestry University, Hangzhou, China 311300.

*For correspondence (fax +1 306 975 4839; e-mail: Adrian.cutler@nrc.ca).

This PDF file includes:

Additional file 1: Figures S1 to S4 and Tables S2, S3 and S5
Methods

Other Supporting Online Materials for this manuscript includes the following:

Additional file 2: Table S1. Significant Gene List Obtained from T-Test P-Value Cut-Off ≤ 0.05 and Fold Change ≥1.5 (Excel File).

Additional file 3: Table S4. MYBR1 Represses Genes Induced by Natural Leaf Senescence (Excel File).

Supporting Online Materials

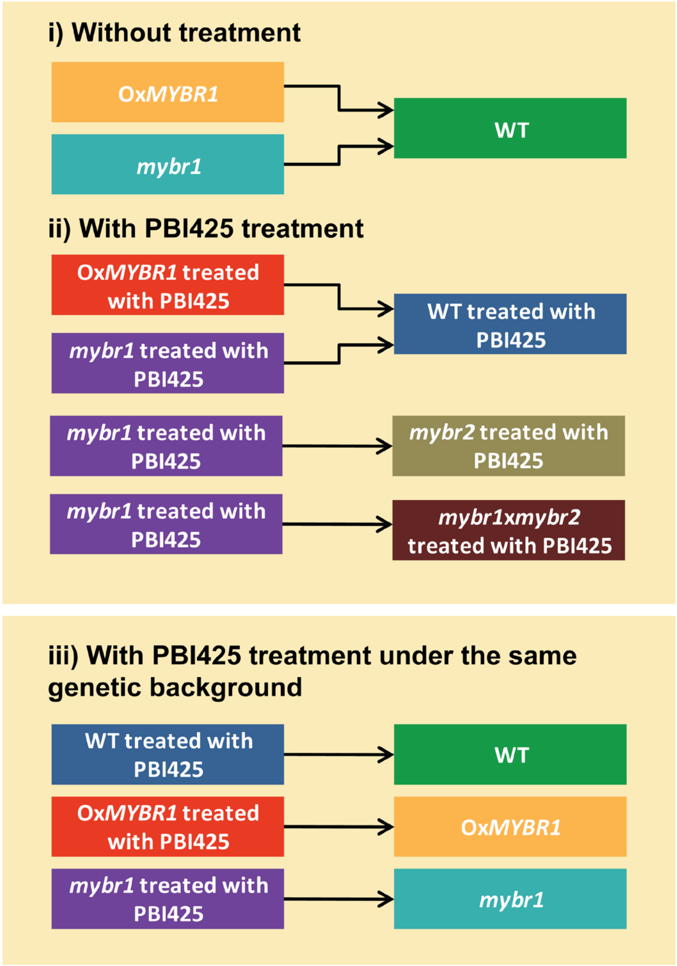

Additional file 1: Figure S1 Experimental Designs of Two Color Arabidopsis Microarray Experiments Using Above-Ground Tissues of 5 Weeks Old Plants.

i) *Genotype comparisons of untreated plants*: effects of *MYBR1* on gene expression changes were studied by comparing gene expression of gain-of function Ox*MYBR1* versus wildtype (WT) (Col-0) and loss-of-function *mybr1* versus WT.

ii) *Genotype comparisons after PBI425 treatments on each genotype*: (+)-8' acetylene ABA (PBI425) is a hyperactive ABA analog which intensifies and/or prolongs expression of ABA responsive genes and hence is an effective tool to identify weakly and transiently expressed ABA responsive genes (Huang et al., 2007). Therefore, 5 weeks old plants were treated with PBI425 for 24 hours and the effects of *MYBR1* on gene expression were studied in comparisons of Ox*MYBR1* (treated with PBI425) versus WT (treated with PBI425) and *mybr1* (treated with PBI425) versus either WT, *mybr1* or *mybr1*X*mybr2* (treated with PBI425).

iii) *Effect of PBI425 treatments on each genotype*: Effects of *MYBR1* on gene expression were studied by treating each genotype (WT, Ox*MYBR1* and *mybr1*) with PBI425 and comparing their gene expressions with untreated of the same genetic background.

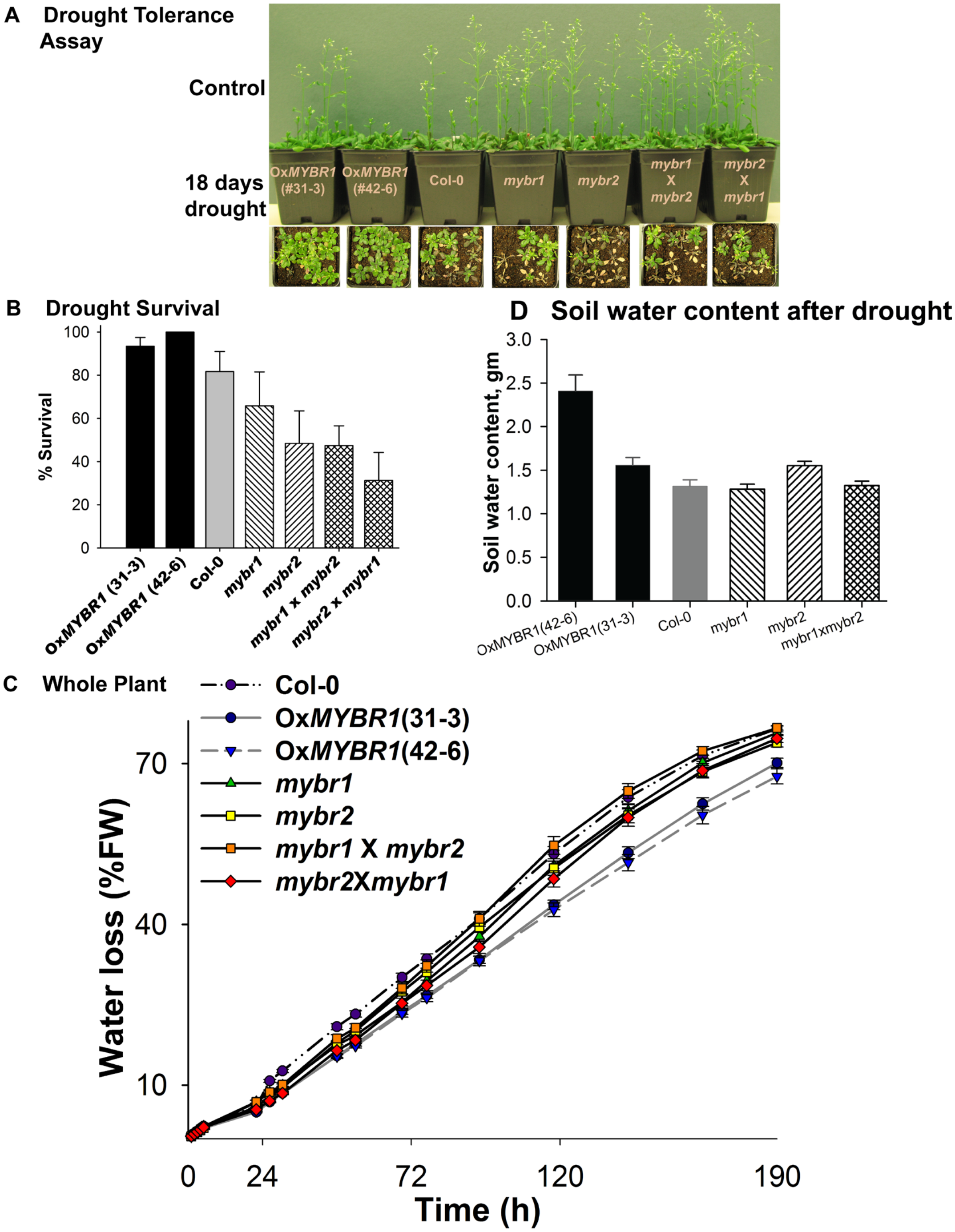

Additional file 1: Figure S2 Reduced Water Uptake by Gain of At*MYBR1* Function Leads to Apparent Drought Tolerance. **(A)** Plants from two 35S::*MYBR1* lines -- 31-3 and 42-6 were used. One-week old seedlings were subjected to drought stress for 18 days by withholding water and ten pots of plants for each genotype were subjected to the stress. Pictures were taken 2 days after rewatering. Control plants were watered regularly as needed. **(B)** Increased survival of drought stress by gain-of-function At*MYBR1* lines -- 31-3 and 42-6. Survival rate and standard error (bar; n=10) were calculated 2 days after rewatering. The P-value between groups of single factor ANOVA is 6.84E-05. **(C)** Transpirational water loss from 24 d old whole plants. Plants were grown normally for 24 d then pots were sealed at the soil surface (plastic wrap) to eliminate evaporation. The pots were weighed at intervals for 190 h. Water loss and standard error were calculated at each time point (bar; n=10). The P-value of two factor ANOVA is 4.2E-98. **(D)** Soil water content after 8 days of drought; standard error (bar; n=12).

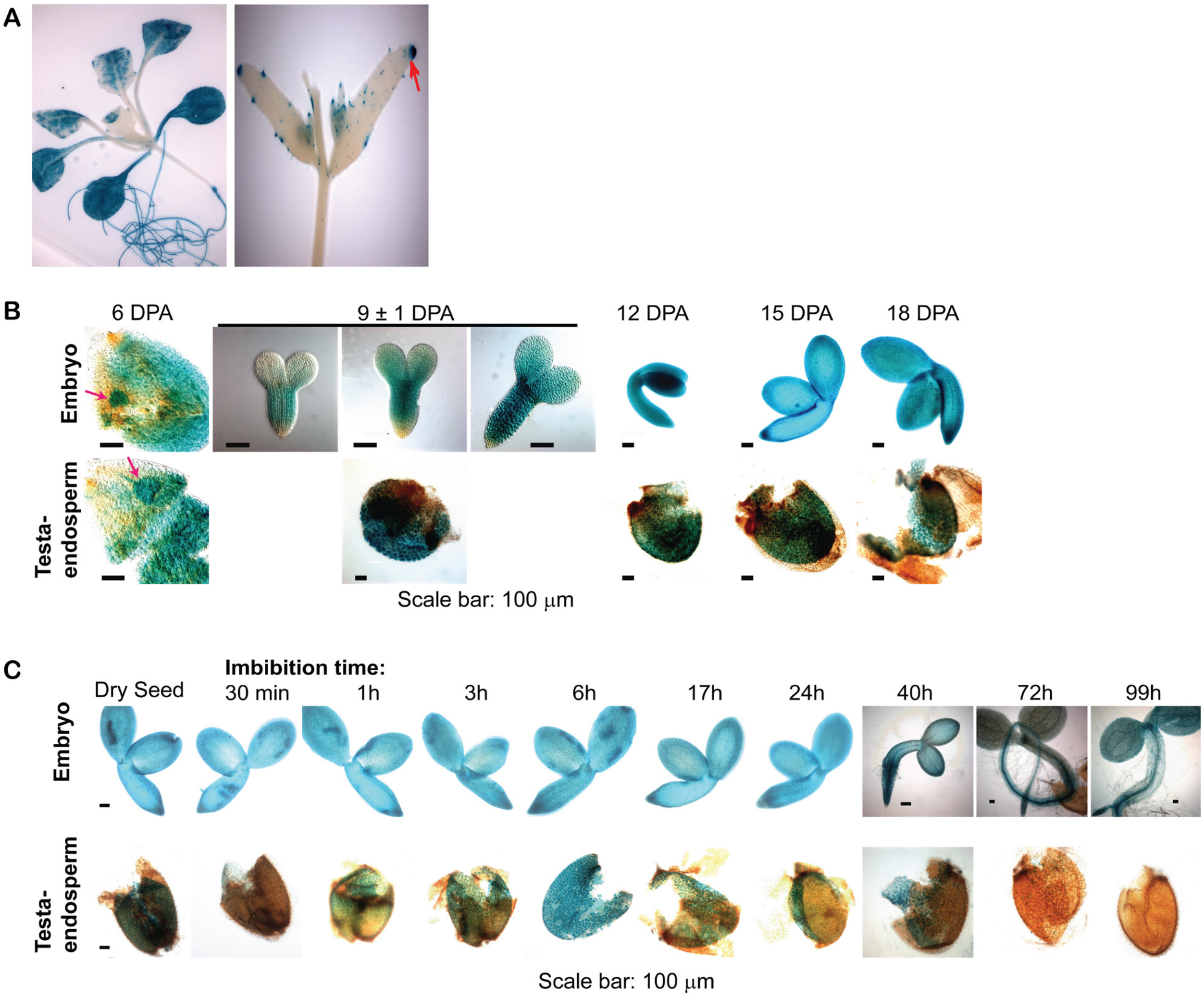

Additional file 1: Figure S3 Expression of *MYBR1*pro:*GUS* in Vegetative Tissues, Embryos and Endosperm at Different Developmental Stages and after Imbibition of Mature Seeds. Histochemical localization of GUS activity was performed by staining with X-gluc for different time intervals as described below and in Methods.

(A) Mature rosette leaves of a 13d old seedling showed more *GUS* expression than juvenile rosette leaves. Very little to no *GUS* expression in cauline leaves except in hydathodes (shown by an arrow) which show intense *GUS* expression.

(B) Developmental expression of *MYBR1*pro:*GUS* in embryos and endosperm collected from siliques at 6-18 DPA. Arrowheads indicate embryos at 6 DPA.

(C) Time course of *MYBR1*pro:*GUS* expression in embryo, endosperm and seedling after imbibition of mature seeds.

Additional file 1: Figure S4 Physical Interaction of MYBR1 and MYBR2 with PYL8, MYBR2 and INO in the Presence of 10 µM of Various Hormones and Inhibitors of Auxin Signaling (2(P-chlorophenoxy)2-methyl propanoic acid; PCIB) and Auxin Transport (2,3,5-triiodobenzoic acid; TIBA and N-(1-naphthyl phthalamidic acid/naptalam; NPA).

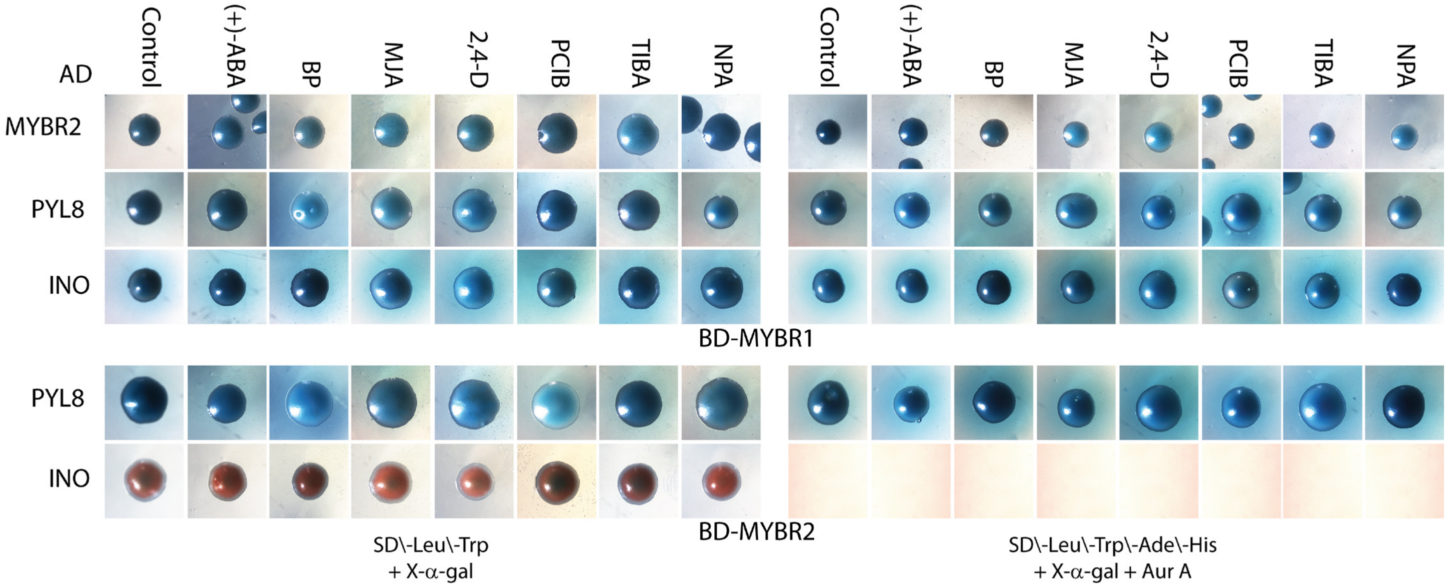

Additional file 1: Table S2 Enriched TF sites (http://www. bioinformatics2.wsu.edu/cgi-bin/Athena/cgi/home.pl and http://arabidopsis.med.ohio-state.edu/AtcisDB/)

P-value	Name of TF binding site motif	Responsive to stimuli	#P	#S
**Repression of ABA response:**				
< 10 - 10	ABRE-like binding site motif	ABA; dehydration; low temperature	106	186
< 10 - 10	ACGTABREMOTIFA2OSEM	ABA	82	121
< 10 - 10	CACGTGMOTIF	ABA; light; UV-light;	72	190
< 10 - 10	GADOWNAT	GA	51	70
< 10 - 6	Z-box promoter motif	Light	19	20
< 10 - 7	ABRE binding site motif	ABA	30	31
< 10 - 4	ABREATRD22	ABA; dehydration	16	16
< 10 - 4	GBOXLERBCS	Light	16	17
< 10 - 4	ABFs binding site motif	ABA	19	20
**Constitutive repression of ABA responses:**				
< 10 - 9	DREB1A/CBF3	Drought; salt; freezing	15	23
< 10 - 8	ABRE-like binding site motif	Dehydration; low temperature	23	42
< 10 - 6	DRE core motif	Drought; high salinity; cold	21	35
< 10 - 5	ACGTABREMOTIFA2OSEM	ABA	17	25
< 10 - 5	CACGTGMOTIF	Light	17	44
< 10 - 5	GADOWNAT	GA	13	15
< 10 - 4	EveningElement promoter moti	Circadian clock	10	12
< 10 - 4	Z-box promoter motif	Light	7	7
**ABA-like repression:**				
< 10 - 5	UPRMOTIFIIAT	Stress	29	30
**MYBR1 independent ABA repression:**				
< 10 - 4	Ibox promoter motif	Light	157	206
**MYBR1 independent ABA activation:**				
< 10 - 10	ABFs binding site motif	ABA	28	31
< 10 - 10	ABRE binding site motif	ABA	35	40
< 10 - 10	ACGTABREMOTIFA2OSEM	ABA	93	148
< 10 - 10	CACGTGMOTIF	Light	70	182
< 10 - 9	ABRE-like binding site motif	Dehydration; low temperature	111	201
< 10 - 9	GADOWNAT	GA	59	81
< 10 - 7	GBOXLERBCS	Light	22	24
< 10 - 5	GBF1/2/3 BS in ADH1	Light	14	28
< 10 - 4	ABREATRD22	ABA	16	16
< 10 - 4	TGA1 binding site motif	Light	19	22
< 10 - 4	UPRMOTIFIAT	Stress	19	22
< 10 - 4	Z-box promoter motif	Light	17	18

#P: number of promoters w ith TF sites; **#S**: number of predicted TF sites

Additional file 1: Table S3 Primers for QRT-PCR and Gene Cloning for Yeast Two-Hybrid and BiFC Analysis.

Gene	AGI	Primers (5' → 3')
**QRT-PCR**		
ACTIN2	At3g18780	F: aacccaaaggccaacagaga
		R: aaggtcacgtccagcaaggt
MYBR1	At5g67300	F: tctccacctgttgttactgggctt
		R: ttgactcgtggctacggtttgact
SAG12	At5g45890	F: gctgatttaaccaatgacga
		R: tttcttcctccagtcaacag
SAG21	At4g02380	F: gaactctccaatgctatcttcc
		R: ggttgattcttccactccct
SAG29	At5g13170	F: tcacgctttctttcttcctc
		R: ggactcatcacgacaatactc
SEN1	At4g35770	F: ctggtcatcggctatttctc
		R: catttcctctgcttgttgtc
SEN4	At4g30270	F: ctctccttagacaaatcctctg
		R: ccactcatattacccaagaactc
ABI5	At2g36270	F: cggtgtcttcagatggatta
		R: attccactgtatatgcttgttt
EEL	At2g41070	F: gagacagtagtccctcaaga
		R: ctctaattcatgtgtataagcctg
ABF1	At1g49720	F: gggtttatttgagactagcg
		R: atcttgattcactagcttgagac
ABF2	At1g45249	F: attgtcatcagaagggatagg
		R: ctgctttcgttgtaactcgt
ABF3	At4g34000	F: ctgatctgactccaaagagc
		R: ttccagttccatcgtataagc
ABF4	At3g19290	F: caggtctagagaaggttatcg
		R: ttccagttccaatgtataagcc
**Gateway cloning of ORFs into pGADT7 vector - Yeast two-hydrid**		
PYR1	At4g17870	F: ggggacaagtttgtacaaaaaagcaggcttaatgccttcggagttaaca
		R: ggggaccactttgtacaagaaagctgggtacgtcacctgagaaccact
PYL1	At5g46790	F: ggggacaagtttgtacaaaaaagcaggctgcatggcgaattcagagtcctc
		R: ggggaccactttgtacaagaaagctgggtacctaacctgagaagagttgttgt
PYL2	At2g26040	F: ggggacaagtttgtacaaaaaagcaggcttaatgagctcatccccggcc
		R: ggggaccactttgtacaagaaagctgggtattattcatcatcatgcatagg
PYL3	At1g73000	F: ggggacaagtttgtacaaaaaagcaggcttaatgaatcttgctccaatccatg
		R: ggggaccactttgtacaagaaagctgggtatcaggtcggagaagccgtgga
PYL5	At5g05440	F: ggggacaagtttgtacaaaaaagcaggcttaatgaggtcaccggtgcaa
		R: ggggaccactttgtacaagaaagctgggtattgccggttggtacttcg
PYL6	At2g40330	F: ggggacaagtttgtacaaaaaagcaggctgcatgccaacgtcgatacagt
		R: ggggaccactttgtacaagaaagctgggtacgagaatttagaagtgttctcg
PYL7	At4g01026	F: ggggacaagtttgtacaaaaaagcaggctgcatggagatgatcggaggaga
		R: ggggaccactttgtacaagaaagctgggtaaaggttggtttctgtatgattct
PYL8	At5g53160	F: ggggacaagtttgtacaaaaaagcaggcttaatggaagctaacgggattgaga
		R: ggggaccactttgtacaagaaagctgggtattagactctcgattctgtcgt
PYL9	At1g01360	F: ggggacaagtttgtacaaaaaagcaggcttaatgatggacggcgttgaa
		R: ggggaccactttgtacaagaaagctgggtactgagtaatgtcctgaga
PYL10	At4g27920	F: ggggacaagtttgtacaaaaaagcaggctgcatgaacggtgacgaaacaaaga
		R: ggggaccactttgtacaagaaagctgggtatatcttcttctccatagattctgcttgt

PYL11	At5g45860	F: ggggacaagtttgtacaaaaaagcaggcttaatggaaacttctcaaaaatatc<br>R: ggggaccactttgtacaagaaagctgggtattacaactttagatgagccac
PYL12	At5g45870	F: ggggacaagtttgtacaaaaaagcaggcttaatgaaaacatctcaagaacag<br>R: ggggaccactttgtacaagaaagctgggtattaagtgagctccatcatctt
PYL13	At4g18620	F: ggggacaagtttgtacaaaaaagcaggcttaatggaaagttctaagcaaaaacgatg<br>R: ggggaccactttgtacaagaaagctgggtattacttcatcattttctttgt
**In-Fusion cloning of ORFs into pGBT9 vector - Yeast two-hydrid**		
MYBR1	At5g67300	F: tgtatcgccggaattcatggctgataggatcaaaggtcca<br>R: gcaggtcgacggatccctactcgattctcccaactccaatttg
MYBR2	At3g50060	F: tgtatcgccggaattcatggcggatcgtgttaaagg<br>R: gcaggtcgacggatccctactcaaccttaggtgttattactcc
**Gateway cloning of ORFs into pDONR221 vector - BiFC**		
MYBR1	At5g67300	F: ggggacaagtttgtacaaaaaagcaggcttaatggctgataggatcaaa<br>R: ggggaccactttgtacaagaaagctgggtactcgattctcccaactccaattt
MYBR2	At3g50060	F: ggggacaagtttgtacaaaaaagcaggcttaatggcggatcgtgttaaa<br>R: ggggaccactttgtacaagaaagctgggtactcaaccttaggtgttat
PYL8	At5g53160	F: ggggacaagtttgtacaaaaaagcaggcttaatggaagctaacgggattga<br>R: ggggaccactttgtacaagaaagctgggtagactctcgattctgtcgtgtctt

F, forward; R, reverse

Additional file 1: Table S5 *MYBR1* represses genes associated with leaf senescence. Microarray data of present study and van der Graaff et al. [21] were compared and gene regulation of common genes between the two studies is listed. NS: developmental senescence; DIS: darkening-induced senescence; DET: senescence in dark-induced detached leaf; Gene regulation symbols ↑: upregulation; ↓: downregulation; —: either 'not differentially expressed' or 'undetectable'. Number of genes in bold font constitutes 70% or more of the common genes and asterisk represents 90% or more.

			**Gene regulation (N^o gene)**				
			Present study		*van der Graaff et al.*		
**Class**	**Classification of gene regulation type**	**N^o common gene**	**ABA**	**MYBR1**	**NS**	**DIS**	**DET**
**A**	Repression of ABA response	165	↑ (165)	↓ (165)	**↑(145)**<br>↓(19)	**↑(123)**<br>↓(34)	↑(101)<br>↓(55)
**B**	ABA-like repression	161	↓ (161)	↓ (161)	↑(16)<br>**↓(144)**	↑(9)<br>**↓(150)***	↑(10)<br>**↓(150)***
**C**	MYBR1 independent ABA repression	261	↓ (261)	—	↑(13)<br>**↓(248)***	↑(8)<br>**↓(251)***	↑(11)<br>**↓(249)***
**D**	ABA independent repression	59	—	↓ (59)	↑(13)<br>**↓(45)**	↑(11)<br>**↓(48)**	↑(12)<br>**↓(45)**
**E**	Constitutive activation of ABA-repressed responses	10	↓ (10)	↑ (10)	↑(1)<br>**↓(9)***	**↓(10)***	**↓(9)***
**F**	ABA-like activation	38	↑ (38)	↑ (38)	**↑(27)**<br>↓(11)	↑(18)<br>↓(17)	↑(18)<br>↓(19)
**G**	MYBR1 independent ABA activation	146	↑ (146)	—	**↑(126)**<br>↓(19)	**↑(116)**<br>↓(26)	↑(98)<br>↓(45)
**H**	ABA independent activation	11	—	↑ (11)	↑(7)<br>↓(4)	↑(1)<br>**↓(9)**	↑(1)<br>**↓(9)**

Additional Methods

Drought Tolerance Assay

Drought tolerance assay was performed as follows: 3.5 inch square pots were filled with wet soil (Sunshine 3 Mix from Sun Gro Horticulture Inc.) in ten replicates for each genotype. Seeds of each genotype were sprinkled on soil, and pots were equilibrated with water for three days during stratification at 4 ^{o}C. Pots were transferred to a growth chamber at 22°C and 40% humidity with 16 h of 165 µE light and 8 h dark cycles and covered with transparent cover for 2 days. Four days after transfer to growth chamber, excess seedlings were discarded leaving 8-11 well-spaced seedlings per pots. On day 7 of transfer, drought stress was imposed by withholding water for 18 days. Data was collected and pictures were taken 2 days after re-watering. Controls were watered regularly at 3 days interval.

Transpirational Water Loss Assay of Whole Plants

Seeds were sewn densely as described to create a bush of foliage covering the soil. On day 24, watering was stopped and pots were sealed with several layers of cling wrap to minimize water loss other than from plants. Pots were weighed at intervals up to 190 h. A fan was placed in the growth chamber to circulate air uniformly.

Soil Water Content Measurement after Drought

Following stratification at 4 ^{o}C plants were grown in soil for 15 d in a growth chamber of 22°C and 64% humidity with 16 h of 150 µE light and 8 h dark cycles then transplanted individually into 2"x 2.5" pots filled with 90 ml sand: soil (2:1) mix and each pot was watered with 30 ml Hoagland solution. Plants were watered again after 12 d as above. After 8 d, aerial parts of plants were discarded and soil weight was measured. Then soil was dried for 18 h at 110 ^{o}C and soil weight was measured again. Water content was calculated from the difference of soil dry and wet weight.
